# Medication administration in aged care facilities: A mixed‐methods systematic review

**DOI:** 10.1111/jan.16318

**Published:** 2024-07-07

**Authors:** Stephanie Garratt, Alison Dowling, Elizabeth Manias

**Affiliations:** ^1^ School of Nursing and Midwifery Monash University Melbourne Australia

**Keywords:** clinical decision‐making, communication, gerontology, medication, medication management, nurse ‐ patient interaction, nursing home care, older people, systematic reviews and meta‐analyses

## Abstract

**Aim(s):**

To synthesize aged care provider, resident and residents' family members' perspectives and experiences of medication administration in aged care facilities; to determine the incidence of medication administration errors, and the impact of medication administration on quality of care and resident‐centredness in aged care facilities.

**Design:**

A mixed‐methods systematic review. PROSPERO ID: CRD42023426990.

**Data Sources:**

The AMED, CINAHL, MEDLINE, EMBASE, EMCARE, PsycINFO, Scopus and Web of Science core collection databases were searched in June 2023.

**Review Methods:**

Included studies were independently screened, selected and appraised by two researchers. The Preferred Reporting Items for Systematic Reviews and Meta‐Analysis (PRISMA) checklist was followed, with the Mixed Methods Appraisal Tool was used for critical appraisal. Convergent synthesis of data, thematic synthesis and meta‐analysis were performed.

**Results:**

One hundred and twenty‐eight studies were included (33 qualitative, 85 quantitative and 10 mixed‐methods). Five themes were formulated, including 1) Staffing concerns, 2) The uncertain role of residents, 3) Medication‐related decision‐making, 4) Use of electronic medication administration records and 5) Medication administration errors. Educational interventions for aged care workers significantly reduced medication administration errors, examined across five studies (OR = 0.37, 95%CI 0.28–0.50, *p* < .001).

**Conclusions:**

Medication administration in aged care facilities is challenging and complex on clinical and interpersonal levels. Clinical processes, medication errors and safety remain focal points for practice. However, more active consideration of residents' autonomy and input by aged care workers and providers is needed to address medication administration's interpersonal and psychosocial aspects. New directions for future research should examine the decision‐making behind dose form modification, aged care workers' definitions of medication omission and practical methods to support residents' and their family members' engagement during medication administration.

**Implications for the profession and/or patient care:**

It is important that medication administration in aged care facilities be more clearly acknowledged as both a clinical and interpersonal task. More attention is warranted regarding aged care workers clinical decision‐making, particularly concerning dose form modification, covert administration and medication omissions. Resident‐centred care approaches that support resident and family engagement around medication administration may improve adherence, satisfaction and quality of care.

**Impact:**

*What Problem Did the Study Address?* Medication administration in aged care facilities is a complex clinical and interpersonal activity. Still, to date, no attempts have been made to synthesize qualitative and quantitative evidence around this practice. There is a need to establish what evidence exists around the perspectives and experiences of aged care workers, residents and resident's family members to understand the challenges, interpersonal opportunities and risks during medication administration.

*What Were the Main Findings?* There is a lack of empirical evidence around resident‐centred care approaches to medication administration, and how residents and their families could be enabled to have more input. Dose form modification occurred overtly and covertly as part of medication administration, not just as a method for older adults with swallowing difficulties, but to enforce adherence with prescribed medications. Medication administration errors typically included medication omission as a category of error, despite some omissions stemming from a clear rationale for medication omission and resident input.

**Where and on Whom Will the Research Have an Impact?:**

The findings of this systematic review contribute to aged care policy and practice regarding medication administration and engagement with older adults. This review presents findings that provide a starting point for aged care workers in regards to professional development and reflection on practice, particularly around clinical decision‐making on dose form modification, medication administration errors and the tension on enabling resident input into medication administration. For researchers, this review highlights the need to develop resident‐centred care approaches and interventions, and to assess whether these can positively impact medication administration, resident engagement, adherence with prescribed medications and quality of care.

**Reporting Method:**

This systematic review was reported in accordance with the Preferred Reporting Items for Systematic Reviews and Meta‐Analyses (Page et al., 2021).

**Patient or Public Contribution:**

No patient or public contribution to this systematic review.

**What does this paper contribute to the wider global clinical community?**

- There is a lack of empirical evidence around resident‐centred care approaches to medication administration, and how residents and their families could be enabled to have input on medication administration.
- Dose form modification occurs overtly and covertly as part of medication administration, not just as a method for older adults with swallowing difficulties, but to enforce adherence with prescribed medications.
- Medication administration errors are largely made up of wrong time errors and omissions, and are more likely to occur during morning medication rounds. Medication omission is typically considered a form of error, despite some omissions stemming from a clear rationale for medication omission and resident input.

## 1 INTRODUCTION

Medication administration is a clinical activity conducted in aged care facilities, where aged care workers provide residents with medications prescribed for treating, diagnosing or preventing an illness or condition (Wilson et al., 2010). The medication administration process involves preparing and delivering medication to older adults, otherwise known as residents (Sefidani Forough et al., 2018; Wilson et al., 2010). Medication administration requires clinical decision‐making and collaboration between aged care workers, residents and their families.

Medication administration involves aged care workers carefully adhering to each resident's medication chart, ensuring that the right resident receives the right medication at the right time, at the right dosage, via the correct route—all documented correctly (Grissinger, 2010). However, aged care workers such as certified nursing staff, or unregulated staff, for example, care assistants, often have competing demands, professional role and workload pressures, on top of time constraints to complete clinical and personal care tasks (Oppert et al., 2018; Papastavrou et al., 2014). These demands and pressures require decision‐making and care prioritizing, which may lead to medication administration errors or dose omissions (Hughes, 2008; Ludlow et al., 2021; Papastavrou et al., 2014).

Common medication administration error types include wrong dosage and dose omission (Crespin et al., 2010; Ferrah et al., 2017; Taxis & Quoc, 2011) In their 2011 systematic review, Taxis and Quoc found that medication administration error rates in aged care facilities varied from 4% to 31%. Adverse drug events, which relate to residents experiencing harm from medication exposure, are commonly associated with medication administration errors (Dimant, 2001; Greene et al., 2010). Al‐Jumaili and Doucette (2018) reported a rate of 6.13 adverse drug events per 100 residents per month, with 4.5% leading to hospitalization and 3.6% leading to an emergency department presentation. Errors or delays during medication administration may prompt residents and their family members to lodge complaints about quality of care, especially regarding pain management or medications for Parkinson's disease (Breen et al., 2023).

Older adults in aged care facilities may also experience challenges with swallowing oral medications, requiring aged care workers to modify doses and administer these in medication delivery vehicles such as food or drink (Haw & Stubbs, 2010; Sefidani Forough et al., 2018). However, modifying dose forms for medication administration can be motivated by factors apart from swallowing, including resident refusal of medication and difficulties with administering medications on time (Haw & Stubbs, 2010; Sefidani Forough et al., 2018). Haw and Stubbs' (2010) systematic review found that modifying dose forms led to medication administration errors, resident harm and variable dosage if the medication was not administered correctly. Covert medication administration, which is the decision by aged care workers to modify doses and administer medications in food or beverages without resident knowledge, often takes place without consulting the multidisciplinary care team. Haw and Stubbs (2010) reported that 1.5%–17% of residents experienced this phenomenon and that decisions to administer medication covertly were poorly documented in practice. There remains a need to reduce ambiguity around dose form modification and covert administration as responses to medication refusal, as well as staffing constraints during medication administration (Sefidani Forough et al., 2018). Since the Haw & Stubbs, 2010 review, there have been a large number of studies published regarding dose form modification in aged care facilities (McDerby et al., 2019; McGillicuddy et al., 2016; Mercovich et al., 2014; Sefidani Forough et al., 2020a), but no subsequent reviews that critically appraised this research.

Prior systematic reviews have focussed on the clinical rather than psychosocial aspects of medication administration, such as pharmacy‐led interventions, adverse drug events or dose form modification in aged care facilities (Ali et al., 2021; Chen et al., 2019; Thiruchelvam et al., 2017). The exception is Manias et al. (2021), who reviewed resident and family communication about medication management in aged care facilities. No systematic reviews have focussed on medication administration in aged care from both psychosocial and clinical aspects, and none have included a synthesis of qualitative study findings alongside quantitative and intervention studies.

## 2 THE REVIEW

### 2.1 Aim(s)

This mixed‐methods systematic review aims to examine research evidence around medication administration in aged care facilities.

Two research questions are addressed: (i) what are the aged care provider, resident and residents' family members' perspectives and experiences of medication administration activities; and (ii) what is the impact of medication administration interventions in aged care facilities on resident‐centred care and quality of residents' care?

### 2.2 Design

The authors adhered to the Preferred Reporting Items for Systematic Reviews and Meta‐Analyses guidelines, and guidance for the conduct of mixed‐methods systematic reviews (Lizarondo et al., 2020; Page et al., 2021; Stern et al., 2021) (Data S1). The protocol was registered with the International Prospective Register of Systematic Reviews (PROSPERO ID: CRD42023426990).

### 2.3 Search strategy, information sources and eligibility criteria

The review team and a university librarian with expertise in systematic reviews developed the search strategy. The eligibility criteria and search strategy were informed by the PICo (Population, Phenomena of interest, Context) framework (Lockwood et al., 2015). The overarching terms used for the population were ‘family’, ‘caregivers’, ‘health personnel’ and ‘aged’; for the phenomena of interest, these terms were ‘medication systems’ or ‘medication administration’; and for the context, this term was ‘residential facilities’. Search term concepts were combined using Boolean ‘AND’, with Boolean ‘OR’ used to include alternative spelling of terms and synonyms. The AMED, CINAHL, MEDLINE, EMBASE, EMCARE, PsycINFO, Scopus and Web of Science core collection databases were searched up until 9 June 2023. Table 1 presents an example of a search strategy used.

**TABLE 1:** Example of a search strategy (Ovid MEDLINE).

exp residential facilities/ (long term care home* or long term care facilit* or aged care home* or residential aged care facilit* or aged care service* or nursing home*1 or skilled nursing facilit*).mp. 1 or 2 exp Aged/ (nurs* or family or families or sibling* or spouse* or caregiver* or relative* or patient* or resident* or elder* or older adult* or aged or nursing home patient or health personnel or healthcare professional*1 or attitudes of health personnel or health care profession*1 or health care provider* or nursing home personnel or nurses role or nursing staff).mp. 4 or 5 exp Medication Systems/ (medication administration or medication decision* or medication system* or medicine* administration or drug* administration or medication safety or medication compliance).mp. 7 or 8 3 and 6 and 9

Inclusion criteria encompassed research studies published in peer‐reviewed journals, conducted in aged care facilities (or equivalent), and published in English. A range of terms are used to describe aged care facilities, including nursing homes, long‐term care and residential aged care. For this review, we included sites where services provided continuous supported care for older adults who could no longer live in a private dwelling or home. This care included accommodation, personal care, nursing and general healthcare services. As a result, studies set in skilled nursing facilities and assisted living were included if they reported on medication administration processes. No limitations on publication date or study design were set. An open date range was deliberately chosen to allow for an examination of discourses and interventions over time, in the absence of prior reviews focussed on medication administration in aged care facilities. Grey literature and prior reviews (systematic or scoping) were excluded from this review, as were conference abstracts if their corresponding full papers were unavailable.

### 2.4 Data extraction

**FIGURE 1:**
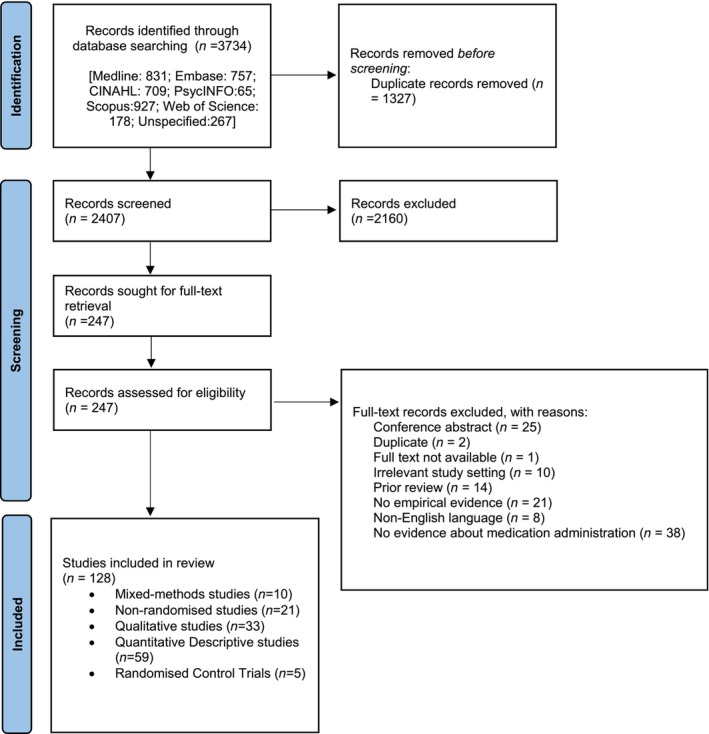
PRISMA 2020 flow chart of study selection.

References were imported into EndNote 20, and then into Covidence review software (Veritas Health Innovation, 2021), where duplicates were removed, and screening and data extraction took place. Two independent reviewers screened study titles and abstracts for relevancy according to the eligibility criteria, with a third reviewer examining and resolving any discrepancies. The screening process is delineated in Figure 1. Relevant papers at the title and abstract level were assessed for eligibility at the full‐text level using two independent reviewers, with any discrepancies resolved by a third reviewer.

Data from the included studies were extracted into a standard form, which included: the first author's name, year of publication, country, study characteristics, participant characteristics, key findings, medication error rates (if relevant) and limitations of each study. For qualitative data, themes and findings around perspectives or experiences of medication administration were extracted. Quantitative data, including results tables, narrative summaries of findings, intervention types and effects, were extracted separately from qualitative data (including mixed‐methods studies). Two reviewers carried out data extraction and critical appraisal processes, with any discrepancies resolved by consensus with input from a third reviewer.

### 2.5 Quality appraisal

The Mixed‐Methods Appraisal Tool was selected to appraise the methodological quality of included studies, in accordance with the Johanna Briggs Manual for Evidence Synthesis (Lizarondo et al., 2020; Stern et al., 2021). This required a minor amendment to the PROSPERO protocol, which originally included separate tools for individual study design types. Use of these tools would not have been appropriate given the final set of included texts. The Mixed‐Methods Appraisal Tool facilitates the appraisal of studies in five overarching designs: randomized control trials, qualitative, mixed‐methods, quantitative descriptive and non‐randomized studies (Hong et al., 2018). The Mixed‐Methods Appraisal Tool was also used to assess risk of bias (Hong et al., 2018). Two reviewers conducted the quality appraisal independently, and later met to discuss discrepancies. A third reviewer resolved any discrepancies that were unable to be resolved. All studies were included in the systematic review regardless of their score on the Mixed‐Methods Appraisal Tool, as the tool does not set out a cut‐off for inclusion (Table S1).

### 2.6 Synthesis

A convergent segregated design was used with independent quantitative and qualitative data synthesis, followed by integrating the two evidence types (Stern et al., 2021). This approach was taken due to the different dimensions of medication administration in aged care facilities included in this review (experiences/perspectives and effects of interventions) (Stern et al., 2021). Quantitative data from included studies were pooled where possible for the conduct of a meta‐analysis using study designs and outcomes, with relevant data categorized broadly into dichotomous and continuous variables. For dichotomous data, odds ratios were used; for continuous data, mean difference or standardized mean differences were used (Higgins et al., 2023). A statistical meta‐analysis and risk of bias assessment was conducted using RevMan Web (version 6.7.0). The meta‐analysis excluded studies with incomplete data, or if less than two studies used the same type of intervention.

Qualitative data were analysed using thematic synthesis, where data were extracted from included studies, inputted into NVivo software (version 14), read in full and inductive coding was carried out. These codes were then refined further, grouped and labelled to form descriptive themes that were summarized and discussed by the reviewers. These themes are presented in Figure 3 (Thomas & Harden, 2008). Once qualitative and quantitative data were analysed, their synthesized findings were juxtaposed against each other and organized into a configurative analysis (Lizarondo et al., 2020). This configurative analysis involved constant comparison of quantitative and qualitative evidence.

## 3 RESULTS

The database search yielded 3734 studies. Following duplicate removal and screening, 128 studies were included in the final review set (Figure 1; Table S2).

### 3.1 Study characteristics

**FIGURE 2:**
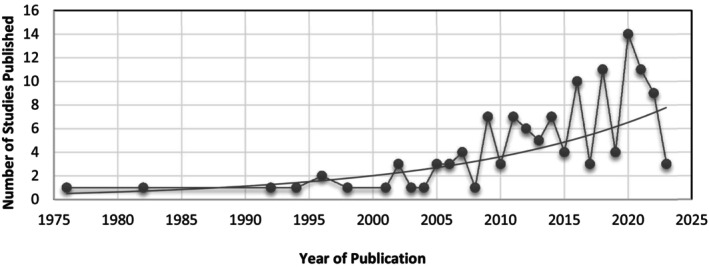
Included study publication dates.

**FIGURE 3:**
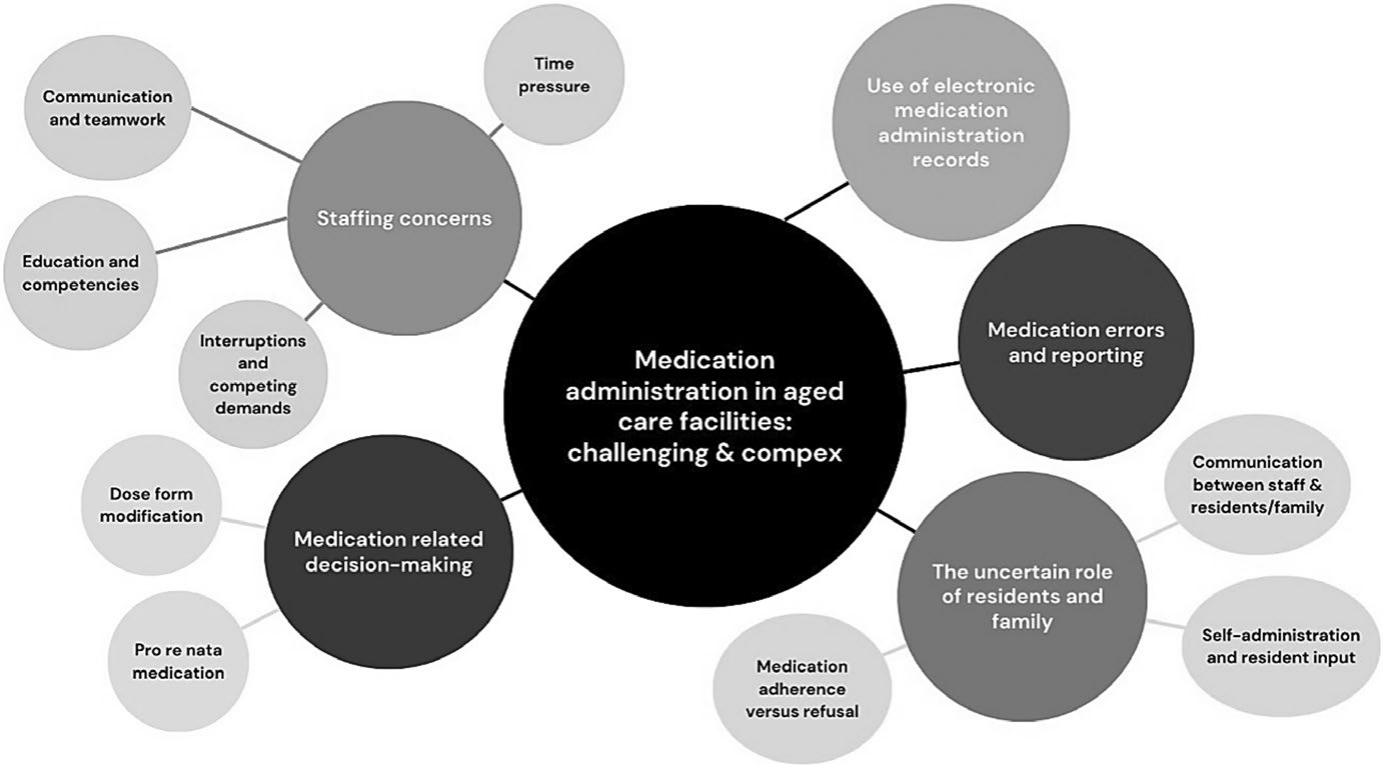
Thematic map of findings.

Included study characteristics are detailed in Figure 3; Table S2. The most common type of study design was quantitative descriptive studies (typically cross‐sectional), comprising 59 studies (46%). Other included studies comprised 33 qualitative descriptive or exploratory studies (26%), 21 non‐randomized studies (16%), 10 mixed‐methods studies (8%) and 5 randomized control trials (4%). Study publication dates ranged from 1976 to 2023, with 63% of studies published between 2013 and 2023 (Figure 2). Several studies from 1976 to 2000 were found to be highly relevant, identifying similar concerns around medication administration and errors compared to studies published between 2013 and 2023. Studies were conducted in 24 countries, with the most prevalent being Australia (*n* = 32 studies), followed by the United States of America (*n* = 28 studies).

Semi‐structured interviews were the most commonly used data collection method for qualitative studies (24 studies), with aged care workers as the most common participant group (31 studies). For mixed‐methods studies, cross‐sectional surveys (six studies), large‐scale audits of medication‐related records (four studies) and interviews (five studies) were used. Of the 85 quantitative studies, 27 used cross‐sectional surveys, and 22 involved auditing or reviewing medication administration‐related records.

In all, 26 studies reported on interventions related to medication administration in aged care facilities, 14 of which were pharmacy‐led, including four of the five randomized‐control trials. Interventions ranged from education of aged care workers (Sanchez et al., 2021; Tenhunen et al., 2014; Verrue et al., 2010), medication regime simplification (Chen et al., 2018; Dugre et al., 2021; Sluggett et al., 2020b; Sluggett et al., 2020a), resident‐centric interventions (Badawoud et al., 2018; Forman et al., 2021) and implementation of electronic medication records (Elliott et al., 2020; Ward et al., 2008; Wild et al., 2011).

### 3.2 Participant characteristics

The included studies spanned 9195 aged care facilities, 599 assisted living sites and 215 skilled nursing facilities. Residents were the most numerous participant type across the included studies, with 135,277 individuals, followed by aged care workers with 7022 individuals. The most common aged care worker type was ‘Registered Nurse’, with 2975 participants. Other licensed aged care workers included Practical Nurses, Enrolled Nurses and Certified Nurse Assistants. The unlicensed staff included in the studies ranged from Nursing Aides or Uncertified Nurse Assistants to Direct Care Workers, Personal Care Assistants and Health Care Assistants. As shown in Table S2, not all studies reported demographic details such as participants' role, age or sex.

### 3.3 Synthesis of results

Medication administration in aged care facilities was a challenging and complex clinical activity; aged care workers needed to complete a sequence of clinical and interpersonal tasks quickly and safely to complete each medication administration. Five main descriptive themes were generated and are presented with supporting illustrative quotes. These themes are: (1) Staffing concerns, (2) The uncertain role of residents and their families, (3) Medication‐related decision‐making, (4) Use of electronic medication administration records technology and (5) Medication errors and reporting (Figure 3).

#### 3.3.1 Theme 1: Staffing concerns

This main theme considers the multifaceted challenges that aged care workers must manage during medication administration in residential aged care facilities. Many of these challenges have persisted for over 30 years, presenting across the included texts regardless of publication date and despite significant changes in how care is delivered in aged care facilities. Registered nurses not only carried out medication administration but also commonly delegated to unlicensed personnel or certified nursing assistants (Alenius & Graf, 2016; Gransjon Craftman et al., 2016; Odberg et al., 2019). Communication and intradisciplinary teamwork were essential, as was time management, given that medication administration was ‘more than a medication round…. it's an everything round’ [Nurse perspective] (Barnes et al., 2006, p. 194).

##### Sub‐theme 1.1. Interruptions and competing demands

Nineteen studies highlighted concerns around interruptions and competing demands on aged care workers' time during medication administration. Interruptions were considered a part of normal practice (Odberg et al., 2018; Zimmerman et al., 2011), and major contributors to medication administration errors (Kuppadakkath et al., 2022; Mahmood et al., 2012; Szczepura et al., 2011).

[…] the phone rings and you have family coming up to you. You need a big sign that says, “Leave me alone” on the cart. Especially when they teach you in school that you shouldn't be interrupted—you should really pay attention to administering [medications]. [Nurse perspective] (Ellis et al., 2012, p. 141)

There was a significant relationship between interruptions and medication administration errors (Scott‐Cawiezell et al., 2007), with rates of interruptions per hour during medication administration varying from 1.2 to 7.2 (Holmqvist et al., 2018; Lee et al., 2015). Interruptions stemmed from aged care workers other than those administering medications, residents, residents' families and emergency alarms (Odberg et al., 2020; Qian et al., 2018; Young et al., 2013). Although residents interrupted, they were also cognisant of the demands on aged care workers, who were ‘[…] too busy doing all sorts of things at once’ [Resident perspective] (Garratt et al., 2021, p. 6).

##### Sub‐theme 1.2. Education and competencies of aged care workers

Aged care workers' competences and ongoing education were reported as facilitators for safe medication administration, noted consistently in studies published across the last 20 years (Eide & Schjøtt, 2001; Kolcu & Ergun, 2020; Lau et al., 2003). Nurses and unlicensed staff rarely received additional, tailored education and training around medication administration, dementia care (Dawud et al., 2022; Sharpp et al., 2012) and dose form modification (Gilmartin et al., 2015; Mercovich et al., 2014; Sefidani Forough et al., 2020b; Tangiisuran et al., 2018). Sites that used unlicensed personnel to assist with medication administration reported heightened concerns from managers and senior registered nurses about aged care workers' ability to administer medication safely (Gransjon Craftman et al., 2016; Hughes et al., 2006; Mitty, 2009).

[…] they have to go to an 8‐hr class […] just on administering medications. These guys are not Registered Nurses and neither am I so I feel like we shouldn't be able to give them, but then again, anyone can read a bottle and do what it says. [Administrator perspective, Assisted Living] (Kemp et al., 2012, p. 6)

##### Sub‐theme 1.3. Communication between aged care workers

Communication was examined across 25 included studies; it could ensure or undermine safe medication administration. Effective communication between aged care workers administering medications fostered trust (Lim et al., 2016; Motta et al., 2018) and accurate verbal and written communication helped aged care workers to work together as a team, especially when they were short‐staffed (Dilles et al., 2011).

It depends on the workload, if our wishes are granted, we have to ensure that no one gets too much to do, that we assign fairly. If we have a nurse on that shift, she will have the final say. Otherwise, it's like the toss of the dice. [Nurse Assistant perspective] (Odberg et al., 2019, p. 388)

##### Sub‐theme 1.4. Time pressures

Time pressure was a key challenge for aged care workers during medication administration, as noted across studies published from 1976 to 2022 (Farner & Hicks, 1976; Hamrick et al., 2007; Kaasalainen et al., 2010; McCloskey et al., 2015; Vogelsmeier et al., 2007). Fourteen studies emphasized time in their results, five were time‐motion studies. Qian et al. (2016) found that nursing staff spent between 22.7% and 29.4% of their time on medication administration, and Chen et al. (2020) found that residents required support on average for 5 min each during breakfast medication administration. For residents, particularly those with pain medication or Parkinson's disease, dose times were critical and delays impacted their quality of life and functional ability (Oates et al., 2019). Ellis et al. (2012) described medication administration using a race metaphor, where nurses would prepare for the medication administration round (the race), carry out the round quickly and accurately (running the race) and then document actions and outcomes (finish the race)

[…] you really have to time‐manage and prioritize…But most importantly, you have to get it done, especially when it comes to meds and treatment […] You have to be quick…otherwise you're there all morning and it's time for the lunchtime meds. [Nurse perspective] (Ellis et al., 2012, p. 138)

However, Kuppadakkath et al. (2022) highlighted that even if aged care workers intended to administer medication on time, this intent may be impossible if they were low staff numbers.

We were only two staff members …it was a busy day, and unfortunately, one day we could not complete our assigned tasks; it was about 1:40 staff‐resident ratio. So, it was very difficult to reach this resident and give the medication at the right time. [Nurse perspective] (Kuppadakkath et al., 2022, p. 882)

#### 3.3.2 Theme 2: The uncertain role of residents and their families

This theme focuses on aged care residents' complex and uncertain role during medication administration. Across studies, residents were described as either compliant or challenging regarding medication administration. For aged care workers, the impetus was to ensure medications were administered quickly and safely. For aged care workers, this meant that ‘sometimes you have to learn little tricks of, okay, how do I get this person to take this? […] It was more just learning about your residents’. [Medication aide perspective] (Carder, 2011, p. 52)

##### Sub‐theme 2.1. Responding to residents' needs

All participant groups noted the importance of aged care workers recognizing that residents were individuals with a lifetime of habits, preferences and needs. Ten qualitative studies referred to the complex needs of residents. These studies typically noted that aged care workers attempted to deliver resident‐centred care during medication administration but experienced difficulties because of residents' diverse needs and preferences (Damiaens et al., 2022; Sawan et al., 2020). The medication administration process took time and planning, involving many residents and small staffing numbers. This limited aged care workers' ability to provide resident‐centric care. Residents themselves were known to interrupt aged care workers out of turn to ensure their needs were met when they preferred, affecting the flow of medication administration rounds (Reinhard et al., 2006; Solberg et al., 2022). To save time, aged care workers would try and pre‐emptively tailor medication administration rounds as much as possible to suit residents.

With nursing home care, you adapt the rounds to suit the patients, you know, they don't have to be rigid…because it's basically the residents' home. [Nurse perspective] (Hughes & Goldie, 2009, p. 510)

##### Sub‐theme 2.2. Adherence versus refusal

Twelve studies were identified as focusing on medication refusal/adherence as a challenge during medication administration. Some residents preferred to have their medication administered to them, taking on a more passive role; they wanted to be adherent with their medication regimen and had struggled to do so when self‐administering (Carder et al., 2009; Oates et al., 2019; Sikma et al., 2014). In addition, improvements in medication adherence were seen through methods that engaged residents actively in their medication administration, with aged care workers' assistance, resident education or mixing medication into palatable preferred mediums (Forman et al., 2021; Park et al., 2013).

I was taking these medicines in a haphazard way at home. I appreciate the routine of the medication schedule and the routine of three‐square meals a day. Sometimes at home I would forget to take my pills for a few days. [Resident perspective] (Carder et al., 2009, p. 469)

Resident adherence to prescribed medication was seen as the ideal by aged care workers—those who refused medication required more time, problem‐solving and persistence, despite many retaining the right to refuse medication (Ailabouni et al., 2017; Garratt et al., 2021; Solberg et al., 2022). Aged care workers sometimes persisted with administration regardless of the residents' decision‐making competence (Barnes et al., 2006; Dilles et al., 2011; Garratt et al., 2021). Medication‐related complaints also noted that residents' right to refuse, preferences and decision‐making could be overlooked or dismissed by aged care workers, or categorized as an error (Breen et al., 2023; Deshmukh & Sommerville, 1996b; Fuller et al., 2022). Reasons for refusal ranged from residents not liking the taste of the medication, being afraid of side effects or deciding they did not need the medication. However, it was more common for studies not to include details about medication refusals, and for residents not to give a reason (Ellis et al., 2012; Garratt et al., 2021; Hughes & Goldie, 2009).

You have to look at what point do we say they have the right to refuse […] It's very challenging […] Some of them [residents] will get smart and be able to lose the pill, without you even noticing that it's not in their mouth, and hide it. [Nurse perspective] (Ellis et al., 2012, p. 141)

##### Sub‐theme 2.3. Resident‐centredness and active engagement

Nineteen studies referred to resident involvement in the medication administration process, largely with self‐administration of medication. This was informed by aged care workers' perspectives and audits of medication records, rather than the perspectives of residents themselves or resident‐centric interventions. Self‐administration by residents was considered a risk by aged care workers, concerned that this level of autonomy could lead to adverse outcomes, adherence issues and medication administration errors (Dube et al., 2018; Prasanna et al., 2020; Vander Stichele et al., 1992). There was an attitude of resignation among residents and families about their ability to participate in medication administration. Also, there was an awareness that their limited involvement may have stemmed from aged care workers' safety concerns, at the cost of resident autonomy and willingness to be involved (Carder et al., 2009; Damiaens et al., 2022; Damiaens et al., 2023).

Everything has to be administered [by staff]. And I find that very disconcerting […] So that takes away independence on my part, you know, it makes me more dependent. [Resident perspective] (Carder et al., 2009, pp. 466‐467)

I would like to be more involved […] Being informed about what [medicines] they are prescribing, […] There is also the question with regard to the administration of medicines […] does he take his medicines? And at what frequency? [Family member perspective] (Damiaens et al., 2023, p. 7)

Two non‐randomized intervention studies involved the examination of self‐administration of medication (Badawoud et al., 2018; Wagner et al., 1994). Counter to commonly reported beliefs around the risks of self‐administration, these studies found that it led to increased resident engagement with their medications (Badawoud et al., 2018), more time for aged care workers to discuss medications with residents (Wagner et al., 1994) and could give residents a sense of ownership over their medication regimen (Solberg et al., 2022). Self‐administration is a large change from medications being administered by aged care workers only, with responsibility and control shifting to residents.

It is so important that the patients can do as much as possible by themselves so that they experience mastering. And taking your own tablets is also a mastery. [Nurse perspective] (Solberg et al., 2022, p. 232)

Promoting resident‐centredness around medication administration did not necessarily require large changes. Two studies used small options for liquids or soft foods for residents to have with their medication (Forman et al., 2021; Hilleary & Ferrini, 2011). In particular, Forman et al. (2021) were concerned with improving the palatability of bitter medication, with residents ranking each option across several measures. These studies provided discrete opportunities to exercise choice, develop preferences and improve adherence, which were not noted to add time or complexity to medication administration for aged care workers (Forman et al., 2021; Hilleary & Ferrini, 2011).

#### 3.3.3 Theme 3: Medication‐related decision‐making

Clinical decision‐making was examined in relation to the administration of Pro Re Nata (PRN) medication (as needed/as required medication) across six studies between 1998 and 2021. Also, the decision about whether to modify a dose form before administering medication to a resident was either a focal point or noted across 22 studies.

##### Sub‐theme 3.1. Pro Re Nata (PRN) medication

PRN medications were common in aged care facilities, with 28%–93% of residents prescribed these medications (Picton et al., 2021; Roberts et al., 1998; Sharma et al., 2021). The most commonly prescribed and administered PRN medications were analgesics and laxatives (Picton et al., 2021; Sharma et al., 2021; Stokes et al., 2004). However, not all aged care workers (licensed or not) in aged care facilities were confident or certified to make the decision to administer PRN medications, and PRN medications added time and complexity to the sequence of medication administration rounds (Carder, 2011; Hughes et al., 2012; Sharma et al., 2021). There was also a significant relationship between PRN medication use and residents having a dementia diagnosis, suggesting that aged care workers may feel more comfortable deciding to administer PRN medication to these residents (Carder, 2011; Stasinopoulos et al., 2018; Stokes et al., 2004). This decision‐making process required aged care workers to assess the resident first, select the right time and dose from the chart and then administer and monitor the resident (Carder, 2011; Tariq et al., 2014).

The last nurse that we had, we had to call her for every PRN medication, just to make sure that we had gone through the proper protocol following the guidelines of whether we could give [a PRN medication] or if we needed to hold off and try, like redirection for behaviours. [Medication Aide perspective] (Carder, 2011, p. 52)

##### Sub‐theme 3.2. Dose form modification

Dose form modification (opening capsules or crushing of tablets and administering doses in another medium, such as yoghurt) was found across studies from the past 20 years, and was identified as common practice during medication administration (McGillicuddy et al., 2016; Sefidani Forough et al., 2020a, 2021; Seifert & Johnston, 2005; Solberg et al., 2021). Not all aged care workers had the knowledge or expertise to decide whether a medication could be safely crushed or a capsule opened (Karttunen et al., 2020; Kirkevold & Engedal, 2009a, 2009b; Wright, 2002). This resulted in inappropriate dose form modification, which sometimes introduced heightened risk of harm to residents (Kirkevold & Engedal, 2010; Mercovich et al., 2014; Paradiso et al., 2002).

It's not about our time, like let's crush them [medications] because it is quicker! It's about what is best for the resident. [Clinical Manager perspective] (Sefidani Forough et al., 2020b, p. 423)

An additional concern was that dose form modification could be conducted covertly; medications were observed or reported as being crushed and hidden in food or beverages without residents' knowledge (Garratt et al., 2021; Paradiso et al., 2002; Solberg et al., 2021). Dose form modification was commonly described as the result of aged care workers' clinical decision‐making, touted as a response to swallowing difficulties (Chen et al., 2020; Jani et al., 2022; Verrue et al., 2011). It was also used in response to residents' non‐adherence with medication regimens or ‘behavioural challenges’ during medication administration (Carvajal et al., 2016; Garratt et al., 2021; Mercovich et al., 2014).

When I give tablets crushed in jam to a patient with dementia, I am used to saying: “Here is your medicine together with some rosehip jam”, and then it's the word “jam” they notice […] …I feel that I'm fooling them, of course, that I am doing something unethical […], but of course we use such methods. [Nurse perspective], (Solberg et al., 2022, p. 233)

#### 3.3.4 Theme 4: Use of electronic medication administration records

The introduction of electronic medication administration records has reduced the time required to carry out medication administration and improved documentation in aged care facilities (Baril et al., 2014; Elliott et al., 2016; Qian et al., 2015; Tariq et al., 2013). Previously, paper‐based records were used, which could be altered after the fact, could be slow to be updated, could be difficult to read and were not securely timestamped (Gilmartin et al., 2014; Lei et al., 2023; Scott‐Cawiezell et al., 2009). These records have also helped to facilitate faster and more detailed medication reviews for residents by pharmacists, which previously required multiple onsite visits for intervention studies (Deshmukh & Sommerville, 1996a; McDerby et al., 2019).

Three intervention studies focussed on the implementation of electronic medication administration records (and their corresponding reminder systems), emphasizing that moving to electronic records reduced medication errors and incidents significantly (Elliott et al., 2020; Fei et al., 2019; Raban et al., 2020).

The advantage of electronic record is to be able to refer to the previous records at any time. For the paper‐based records, we need to look for a long time, and may not be able to find what we need. Sometimes I don't even know where the paper‐based record is, so the electronic record is better. [Nurse perspective] (Lei et al., 2023, p. 8)

The implementation of electronic systems had faced opposition, often from organizations and aged care workers reluctant to move away from traditional paper‐based systems (Karsan et al., 2021; Odberg et al., 2020; Tariq et al., 2014). This reluctance typically stems from concerns over Internet access, the digital literacy skills of the aged care workforce and having to upgrade technology. There were also concerns from nursing staff and aged care administrators that multiple systems could be integrated, resulting in increased documentation and time (Karsan et al., 2021; Tariq et al., 2014).

#### 3.3.5 Theme 5: Medication administration errors and reporting

Medication administration errors were a major concern for aged care workers and residents across the included studies, irrespective of study design. Overall, 47 studies were identified that report specifically on medication error—from identification of error rates to interventions to reduce errors, inconsistent reporting of errors (and why), through to aged care workers' education and stress about errors (Alldred et al., 2011; Gilbert & Kim, 2018; Nicholson & Damons, 2022a, 2022b; van den Bemt et al., 2009). Error reduction and prevention were the primary drivers of interventions in relation to medication administration in aged care facilities. These interventions included the introduction of electronic records, better reporting, higher involvement of pharmacists onsite at aged care facilities and the introduction of new technologies (Karsan et al., 2021; Lei et al., 2023; Vogelsmeier et al., 2022). Incident reports and documentation around errors were identified as inconsistent, and aged care workers feared being punished when an error occurred that may have been prevented (Bengtsson et al., 2021; Garratt, Kerse, Peri, & Jonas, 2020; Lane et al., 2014; Vermeulen et al., 2017).

I've had colleagues who said they did not register the event [medication administration error] because they were afraid […]. [Nurse perspective] (Motta et al., 2018, p. 2485)

Medication administration error rates were reported, albeit in inconsistent formats across 18 studies, primarily those with quantitative descriptive methodologies. Some reported the proportion of dose administrations that were errors, or opportunities for error, which ranged from 7.1% to 42% (Barker et al., 1982; Barker et al., 2002; Santos et al., 2016; Zimmerman et al., 2011). Other studies reported the proportion of residents who experienced a medication administration error during the study timeframe. For example, Barber et al. (2009) and Elliott et al. (2016), found that 22.3% and 24% of residents experienced medication administration errors, respectively.

Medication administration errors were more common during morning medication rounds, which were more complex and challenging for staff compared to other dose times (van den Bemt et al., 2009; Young et al., 2013; Zimmerman et al., 2011). Ten studies specifically reported on wrong time errors, where doses had been administered outside of the time frame indicated on residents' prescriptions, accounting for between 18% and 50.8% of errors (Barker et al., 2002; Breen et al., 2023; Fuller et al., 2022; Nicholson & Damons, 2022a, 2022b; Szczepura et al., 2011). In terms of the causes of medication administration errors, Greene et al. (2005) identified that the primary causes were human factors (53.3% of errors) and communication issues (40.2%). Of the studies, only Al‐Jumaili and Doucette (2018) reported the incidence of adverse drug events, situations where a medication administration error was reported to lead to resident harm. They found a rate of 6.13 adverse drug events per 100 residents per month, with significant positive associations between adverse drug events and a resident having a dementia diagnosis, psychotropic medication, opioids or warfarin (Al‐Jumaili & Doucette, 2018).

Twenty‐five studies reported on medication omission, classifying omission as a type of medication administration error, where medication doses had been prescribed but not administered by the next dose time (Deshmukh & Sommerville, 1996a; Garratt, Kerse, Peri, & Jonas, 2020a; Prasanna et al., 2020). A proportion of medication administration errors were identified as specifically being dose omissions—this ranged from 32% of administration errors to 50.5% (Campagna et al., 2021; Greene et al., 2005; Nicholson & Damons, 2022a, 2022b; Pierson et al., 2007). Similarly to medication administration error rates, Gilmartin‐Thomas et al. (2017) reported an omission rate of 1.6 per 100 doses, versus Garratt, Kerse, Peri, and Jonas (2020) who report a mean rate of 3.59 doses per 100 doses per resident. Only two interrelated studies specified different types of omission: those initiated by residents (refusal of medication) and those by staff (withheld doses, omitted due to clinical decision‐making) (Garratt, Kerse, Peri, & Jonas, 2020; Garratt, Kerse, Peri, Jonas, & Scahil, 2020). Santos et al. (2016) highlight that 58% of medication omissions in their study resulted from clinical decision‐making rather than a mistake or error.

##### Sub‐theme 5.1. Interventions to reduce medication administration error

**FIGURE 4:**
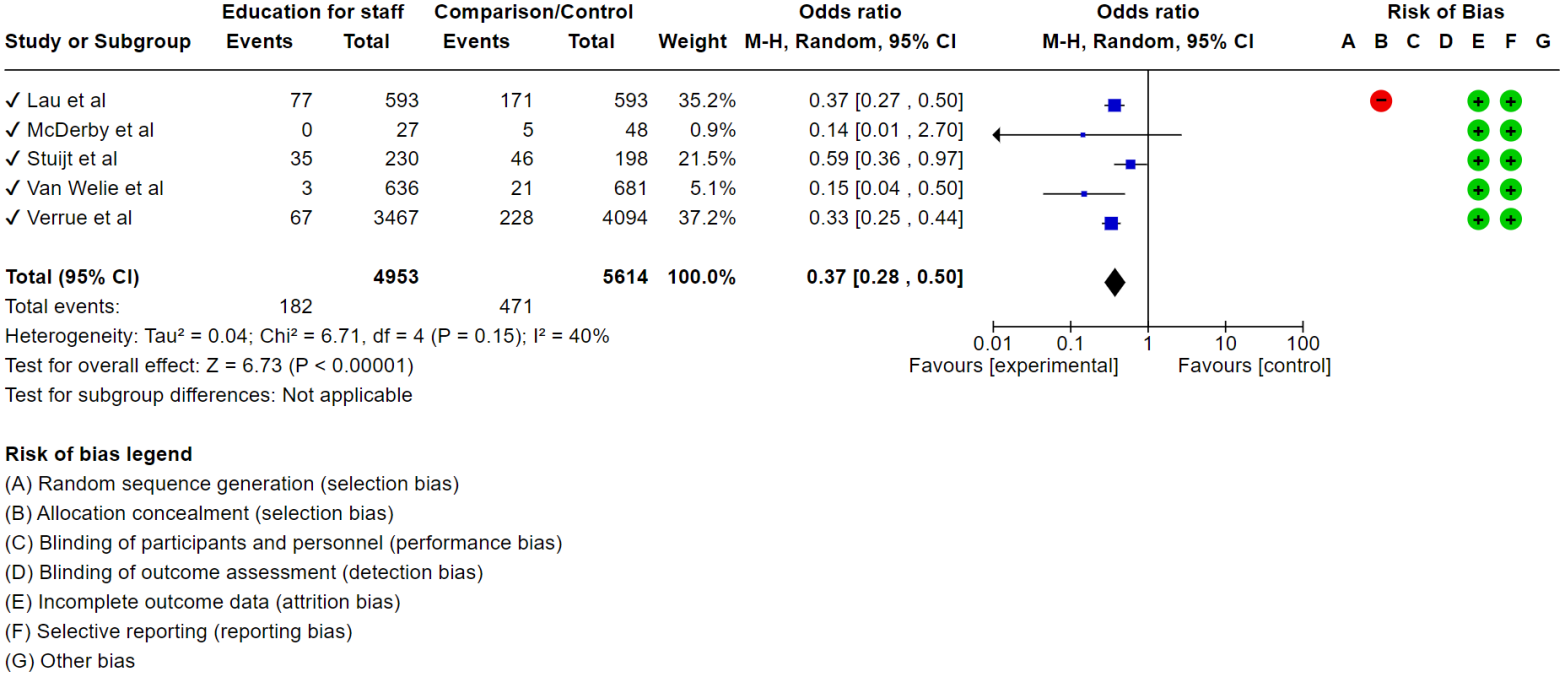
Odds ratio summary and risk of bias summary for studies using education interventions for aged care workers around medication administration errors.

Overall, five studies were eligible for inclusion in a meta‐analysis. All were pharmacy‐led, involving a pharmacist delivering education to aged care workers with the aim of reducing medication administration errors (Lau et al., 2003; McDerby et al., 2019; Stuijt et al., 2013; van Welie et al., 2016; Verrue et al., 2010). This resulted in an odds ratio of 0.37 (95% CI: 0.28–0.50) (Figure 4). Other intervention studies within this systematic review either lacked complete data, or corresponded to an intervention design that was not matched by another study. For risk of bias, aspects A—D and G were largely not applicable as the included studies were non‐randomized (Figure 4). The exception was Lau et al. (2003) which demonstrated a degree of selection bias. For aspects E (attrition bias) and F (reporting bias), green indicated low risk (Figure 4).

## 4 DISCUSSION

This mixed‐methods systematic review provided a comprehensive synthesis of peer‐reviewed evidence around medication administration in aged care facilities, addressing psychosocial and clinical issues. Researchers focused on reducing medication administration errors, ensuring medication adherence, improving clinical processes, educating aged care workers and the perspectives and experiences of aged care workers. Unfortunately, there was a lack of available evidence around residents' and their family members' perspectives and experiences of medication administration, in addition to limited evidence around strategies to support residents' engagement in the administration process. This knowledge gap is of concern, because medication administration is an interpersonal and a clinical activity, and residents may have valuable insights to offer in terms of how this activity could be improved and better support their autonomy as older adults in care. The results of this review indicate that despite almost 50 years of changes to the delivery of care in aged care facilities, challenges remain around staffing, education, clinical decision‐making and involvement of residents and their family members in medication administration.

This review has shown that quantitative studies about medication administration in aged care facilities largely focused on error reduction and medication adherence rather than resident‐centred care in this clinical space. In addition, our analysis identified two areas of high concern in regard to resident input and experience of medication administration—dose form modification and the classification of medication omissions as errors. Finally, there was a lack of well‐design randomized controlled trials on medication administration. Across all intervention studies, half were pharmacist‐led, with medication reviews and aged care worker education being the primary intervention types.

Irrespective of publication date, the studies included in this review primarily focused on medication administration as a clinical task and not necessarily on its interpersonal aspects. Unfortunately, this aligns with the discourse around task‐orientated or ‘production‐line’ care, driven by staffing and time constraints, rather than resident‐centred care (Berkovic et al., 2024; Delaney, 2018; Tuckett, 2005). This has led to a paucity of empirical evidence around resident‐centred care approaches to medication administration in aged care facilities. Resident‐centred care emphasizes partnerships between recipients and providers of care—emphasizing residents'/patients' values and preferences. This systematic review has shown that medication administration is an aspect of everyday life in aged care facilities where the aim is to ‘get the work done’. However, this may seem positive from a clinical perspective, given that aged care workers have a duty of care to residents regarding their medications.

Resident‐centred care approaches to clinical care aspects can positively affect care workers, care recipients and their families (Brownie & Nancarrow, 2013; Park et al., 2018; Santana et al., 2018). There remains a lack of empirical evidence on how aged care facilities can improve their medication administration processes beyond strategies to reduce medication administration errors. These strategies emphasize the role of aged care workers and pharmacists but do little to promote quality resident‐centric care or autonomy. Several studies in this review highlighted the potential of resident‐centred care approaches around medication administration, particularly self‐administration (Badawoud et al., 2018; Solberg et al., 2022; Wagner et al., 1994). However, none of these studies included residents' perspectives, experiences or evaluation of self‐administration. Although these studies had small sample sizes, both identified that supporting residents to self‐administer and engage with their medication administration and enabled aged care workers to spend more quality time with residents rather than persisting with ‘conveyor‐belt’ care (Solberg et al., 2022; Wagner et al., 1994). This contradicts concerns from other studies that outline self‐administration as a safety and error risk (Carder et al., 2009; Dube et al., 2018; Vander Stichele et al., 1992).

It is important to note that less controversial or more discrete interventions could be used to increase resident autonomy and input into medication administration. Giving residents the choice of palatable foods or liquids to have alongside their medication does not require as large a policy and practice change as self‐administration. Still, it allows residents to engage with the administration process. Future interventions could be as simple as allowing residents to choose between flavoured water and normal water with their medications, consulting with residents about dose times or implementing assessments for self‐administration capacity (Forman et al., 2021; Hilleary & Ferrini, 2011; Park et al., 2018). Further research is necessary to establish the feasibility and impact of such resident‐centred care interventions on medication administration from both aged care workers' and residents' perspectives.

This review also showed that dose form modification is more than a response to residents with swallowing issues. Instead, evidence indicated that the decision to modify dose forms could occur frequently, without residents' input, with medication administered covertly to residents to ensure adherence. Dose form modification is primarily promoted as a response to overcome residents' swallowing difficulties with medications, but covert administration of doses without residents' knowledge was also reported as a response to non‐adherence or challenging behaviours (Carvajal et al., 2016; Solberg et al., 2021). This practice could be authorized (approved by members of the broader care team prior), or unauthorized (occurring without collaboration or approval). In both situations, covert administration could occur without the resident's permission. Investigators raised concerns that this practice was undertaken by aged care workers with limited training and knowledge about which medications could be safely modified (Mercovich et al., 2014). Without clear guidance on this practice internationally, dose form modification carries risk of medication administration error and of physical and psychological harm to residents (Garratt et al., 2021; Karttunen et al., 2020; Simpson, 2017).

Covert administration of modified dose forms was considered to be a less invasive and time‐consuming approach to ensuring adherence than verbally or physically coercing residents, notably if they refused medication frequently or lacked the capacity to make decisions around their medication (Ellis et al., 2012; Garratt et al., 2021; Solberg et al., 2022). This weighing up of harm versus benefit regarding clinical outcomes, especially when aged care workers are under time pressure, may make covert administration appear the ‘lesser of two evils’ (Guidry‐Grimes et al., 2021; Munden, 2017). It is important that this is not applied to residents that have capacity to have input into their medication administration and the right to refuse (Kirkevold & Engedal, 2009a). This practice raises ethical and clinical safety concerns, given that its permissibility is unclear on an international scale within aged care facilities, particularly in regard to older adults with fluctuating or unclear capacity (Farrar et al., 2012; Garratt et al., 2021; Young & Unger, 2016).

Our findings also showed that medication omissions continue to be characterized as medication administration errors. This tradition has been maintained from the 1980s to 2023, despite more nuanced understandings around why a dose may be omitted (Garratt, Kerse, Peri, & Jonas, 2020; Santos et al., 2016). In this review, studies typically considered medication omissions to be a form of administration error, irrespective of whether the dose omission had come about due to clinical decision‐making, whether the dose was forgotten by aged care workers, whether the dose had not been delivered yet to the aged care facility or even if the dose was refused by a resident (Fuller et al., 2022; Nicholson & Damons, 2022b). Santos et al. (2016) highlight that of the medication omissions they observed during medication administration, 58% were clinical decisions to omit. This contradicts over 40 years of tradition, where omissions have been broadly defined as an error, first noted in aged care settings by Barker et al. (1982). From an aged care workers perspective, medication omissions, as a form of administration error were typically portrayed negatively as a clinical mistake, risk or failure (Fuller et al., 2022; Nicholson & Damons, 2022b)—which could lead to under‐reporting due to fear of reprisal (Motta et al., 2018). Future studies should consider whether this negative view of omissions from aged care workers stems from education and clinical decision‐making or the association between omissions and errors. Medication omissions also encompassed other scenarios where medication was not administered to residents, including if residents refused medication (Garratt, Kerse, Peri, & Jonas, 2020). Residents may refuse medication for various reasons—to classify these instances as omissions and errors raises ethical concerns, particularly given the clinical focus on adherence and administration error reduction (Gould & Mitty, 2010; Haskins & Wick, 2017). More nuanced reporting would give a clearer picture of why doses are omitted as part of medication administration and whether dose omissions positively or negatively impact clinical outcomes and quality of care.

This review identified a lack of randomized controlled trials focusing on medication administration and an over‐representation of quantitative descriptive or qualitative studies focusing on aged care workers. It was common for data to be either observational or sourced as part of a retrospective review of routinely collected clinical data, methods that are less intensive than a randomized or non‐randomized trial. This review was unable to identify instances where quantitative or qualitative findings were used to directly inform subsequent interventions aimed at improving medication administration in aged care facilities. Educational interventions for aged care workers were identified as effective in reducing medication administration errors—particularly inappropriate dose form modification. These educational interventions were typically delivered by pharmacists, concerned with pharmacotherapy, pharmacology and incident documentation (McDerby et al., 2019; Stuijt et al., 2013). Future research, particularly randomized controlled trial designs that use interventions beyond staff education/pharmacy‐led interventions would be valuable contributions. Additionally, as it becomes more common internationally for aged care facilities to provide a spectrum of care, for example, assisted living, 24‐h nursing care, dementia care and rehabilitation services on one site, it is important for researchers not to exclude these settings. No assisted living or skilled nursing facilities were represented among the intervention studies included in this review. Although these settings have slightly different characteristics, staff and residents experience similar medication administration challenges to aged care facilities, and would benefit from future research which focusses on resident‐centric interventions.

### 4.1 Limitations

This review has several limitations. Only peer‐reviewed studies published in English were included; there may be non‐English or unpublished work that was relevant yet excluded. Conference abstracts were also excluded; there may have been insightful information omitted that was not published in full‐length papers. There may also be relevant empirical research situated in grey literature, which was also excluded as a data source for this review. Regarding the comparison of medication administration rates, this proved difficult due to the heterogenous reporting of rates, frequencies and studies that reported numerators but not denominators.

More restrictive inclusion criteria would not have been advantageous, as earlier reviews have focused on specific interventions that relate to medication errors in aged care broadly, dose form modification or on synthesizing quantitative evidence only. By including all study designs, this review was able to address the importance of staff, residents and residents' family members' perspectives and experiences in addition to clinical concerns.

## 5 CONCLUSIONS

This mixed‐methods systematic review aimed to explore and synthesize evidence around medication administration in aged care facilities, providing a comprehensive understanding of its challenges, complexity and opportunities around resident‐centred care. The findings of this review demonstrated that the perspectives and experiences of aged care workers are given primacy in research, and that interventions to improve medication administration typically focus on pharmacy‐led initiatives or education for aged care workers. Additionally, interventions that did have an impact on resident‐centred care were confined to promoting self‐administration (seen as a risk by aged care workers), or small choices around beverages/soft foods to have with medication. Facilitating more active resident involvement and engagement around medication administration may improve medication adherence and quality of care. This review also showed that clinical decision‐making around medication administration is informed by concerns about medication adherence. In turn, there is now evidence on the adaptation of dose form modification to ensure residents adhere to their medications, undermining residents' autonomy and rights. Medication omissions are seen as a form of error, something to avoid during medication administration, rather than possibly reflecting good clinical decision‐making or resident input. Finally, interventions are also warranted to improve the medication administration process informed by resident‐centred care and to establish whether such interventions can increase resident engagement and adherence.

## AUTHOR CONTRIBUTIONS

**SG, AD and EM**: Made substantial contributions to conception and design, or acquisition of data, or analysis and interpretation of data. **SG, AD and EM**: Involved in drafting the manuscript or revising it critically for important intellectual content. **SG, AD and EM**: Given final approval of the version to be published. Each author should have participated sufficiently in the work to take public responsibility for appropriate portions of the content. **SG, AD and EM**: Agreed to be accountable for all aspects of the work in ensuring that questions related to the accuracy or integrity of any part of the work are appropriately investigated and resolved.

## CONFLICT OF INTEREST STATEMENT

None to disclose.

### PEER REVIEW

The peer review history for this article is available at https://www.webofscience.com/api/gateway/wos/peer‐review/10.1111/jan.16318.

## STATISTICS REPORTING

The authors have checked to make sure that our submission conforms as applicable to the Journal's statistical guidelines.

## Supporting information


Data S1.

Table S1.

Table S2.

## Data Availability

Data sharing is not applicable to this article as no new data were created or analyzed in this study.
