# Supplementary material for: Medication administration in aged care facilities: A mixed‐methods systematic review
Source: J Adv Nurs. 2024 Jul 7;81(2):621–40. doi: 10.1111/jan.16318 (PMC11729541; doi:10.1111/jan.16318)
Supplement: Supplementary file 2 — Table S1. [file JAN-81-621-s001.docx]

|  | | **QUALITATIVE STUDIES** | | | | | | | | | | | | | | | | | | | | | | | | | | | | | | | | |
| --- | --- | --- | --- | --- | --- | --- | --- | --- | --- | --- | --- | --- | --- | --- | --- | --- | --- | --- | --- | --- | --- | --- | --- | --- | --- | --- | --- | --- | --- | --- | --- | --- | --- | --- |
|  | | Barnes et al., 2006 | Bengtsson et al., 2021 | Carder, 2011 | Carder, Zimmerman, & Schumacher, 2009 | Damiaens et al., 2022 | Damiaens, Van Hecke, & Foulon, 2023 | Dawud, Kotecho, & Adamek, 2022 | Ellis et al., 2012 | Garratt et al., 2021 | Gilbert & Kim, 2018 | Gilmartin, Marriott, & Hussainy, 2014 | Gilmartin, Jani, & Smith, 2015 | Gransjon Craftman et al., 2016 | Hughes et al., 2009 | Karsan et al., 2021 | Kuppadakkath, Olasoji, & Garvey, 2022 | Lei et al., 2023 | Motta et al., 2018 | Oates et al., 2019 | Odberg et al., 2018 | Odberg, Hansen, & Wangensteen, 2019 | Odberg et al., 2020 | Qian et al., 2018 | Reinhard et al., 2006 | Sawan, Kouladjian O'Donnell, & Hilmer, 2020 | Sefidani Forough et al., 2020 | Sharpp, Kayser-Jones, & Young, 2012 | Solberg et al., 2022 | Tariq, Georgiou, & Westbrook, 2013a | Tariq, Georgiou, & Westbrook, 2013b | Tariq et al., 2014 | Vogelsmeier, Scott-Cawiezell, & Zellmer, 2007 | Young et al., 2013 |
| **GENERAL QUESTIONS** | Are there clear research questions? | Y | Y | Y | Y | Y | Y | Y | Y | Y | Y | Y | Y | Y | Y | Y | Y | Y | Y | Y | Y | Y | Y | Y | Y | Y | Y | Y | Y | Y | Y | Y | Y | Y |
|  | Do the collected data allow to address the research questions? | Y | Y | Y | Y | Y | Y | Y | Y | Y | Y | Y | Y | Y | Y | Y | Y | Y | Y | Y | Y | Y | Y | Y | Y | Y | Y | Y | Y | Y | Y | Y | Y | Y |
| **QUALITATIVE SCREENING QUESTIONS** | Is the approach appropriate to answer the research question? | Y | Y | Y | Y | Y | N | Y | Y | Y | Y | Y | Y | Y | Y | Y | Y | Y | Y | Y | Y | Y | Y | Y | Y | Y | Y | Y | Y | Y | Y | Y | Y | Y |
|  | Are the data collection methods adequate to address the research question? | Y | Y | Y | Y | Y | Y | Y | Y | Y | Y | Y | Y | Y | Y | Y | Y | Y | Y | Y | Y | Y | Y | Y | Y | Y | Y | Y | Y | Y | Y | Y | Y | Y |
|  | Are the findings adequately derived from the data? | Y | Y | Y | Y | Y | Y | Y | Y | Y | N | Y | Y | Y | Y | Y | Y | Y | Y | N | N | N | N | Y | N | Y | Y | N | Y | Y | Y | Y | Y | N |
|  | Is the interpretation of results sufficiently substantiated by data? | Y | Y | Y | Y | Y | Y | Y | Y | Y | Y | Y | Y | Y | Y | Y | Y | Y | Y | Y | Y | Y | Y | Y | N | Y | Y | Y | Y | Y | Y | Y | Y | N |
|  | Is there coherence between qualitative data sources, collection, analysis and interpretation? | Y | Y | Y | Y | Y | Y | Y | Y | Y | Y | Y | Y | Y | Y | Y | Y | Y | Y | Y | Y | Y | Y | Y | Y | Y | Y | Y | Y | Y | Y | Y | Y | Y |
|  | Total score | 5 | 5 | 5 | 5 | 5 | 4 | 5 | 5 | 5 | 4 | 5 | 5 | 5 | 5 | 5 | 5 | 5 | 5 | 4 | 4 | 4 | 4 | 5 | 3 | 5 | 5 | 4 | 5 | 5 | 5 | 5 | 5 | 3 |

|  | | **RANDOMIZED CONTROL TRIALS** | | | | | **NON-RANDOMIZED STUDIES** | | | | | | | | | | | | | | | | | | | | |
| --- | --- | --- | --- | --- | --- | --- | --- | --- | --- | --- | --- | --- | --- | --- | --- | --- | --- | --- | --- | --- | --- | --- | --- | --- | --- | --- | --- |
|  |  | Dugre et al., 2021 | Forman et al., 2021 | Kolcu & Ergun, 2020 | Sluggett et al., 2020a | Sluggett et al.,2020b | Alenius & Graf 2016 | Badawoud et al., 2018 | Baril et al., 2014 | Carvajal et al., 2016 | Chen et al., 2020 | Deshmukh & Sommerville, 1996 | Eide & Schjott 2001 | Elliott et al., 2020 | Fei, Robinson, & MacNeil, 2019 | Lau et al., 2003 | McDerby et al., 2019 | Park et al., 2013 | Sanchez et al., 2021 | Stuijt et al., 2013 | Tenhunen, Tanner, & Dahlen, 2014 | van Welie et al., 2016 | Verrue et al., 2010 | Vogelsmeier et al., 2022 | Wagner, Wahlberg, & Worning 1994 | Ward et al., 2008 | Wild, Szczepura, & Nelson, 2011 |
| **GENERAL QUESTIONS** | Are there clear research questions? | Y | Y | Y | Y | Y | Y | Y | Y | Y | Y | Y | Y | Y | Y | Y | Y | Y | Y | Y | Y | Y | Y | Y | Y | N | N |
|  | Do the collected data allow to address the research questions? | Y | Y | Y | Y | Y | Y | Y | Y | Y | Y | Y | Y | Y | Y | Y | Y | Y | Y | Y | Y | Y | Y | Y | Y | Y | Y |
| **RANDOMIZED CONTROLLED TRIALS QUESTIONS** | Is randomization appropriately performed? | Y | Y | Y | Y | Y |  |  |  |  |  |  |  |  |  |  |  |  |  |  |  |  |  |  |  |  |  |
|  | Are the groups comparable at baseline? | Y | Y | Y | Y | Y |  |  |  |  |  |  |  |  |  |  |  |  |  |  |  |  |  |  |  |  |  |
|  | Are there complete outcome data? | Y | Y | Y | Y | Y |  |  |  |  |  |  |  |  |  |  |  |  |  |  |  |  |  |  |  |  |  |
|  | Are outcome assessors blinded to the intervention provided? | N | Y | N | Y | Y |  |  |  |  |  |  |  |  |  |  |  |  |  |  |  |  |  |  |  |  |  |
|  | Did the participants adhere to the assigned intervention? | Y | Y | Y | Y | Y |  |  |  |  |  |  |  |  |  |  |  |  |  |  |  |  |  |  |  |  |  |
| **NON-RANDOMIZED STUDIES QUESTIONS** | Are the participants representative of the target population? |  |  |  |  |  | N | Y | N | Y | N | Y | Y | N | Y | Y | N | Y | Y | N | Y | Y | Y | N | N | Y | Y |
|  | Are measurements appropriate regarding both the outcome and intervention (or exposure)? |  |  |  |  |  | Y | Y | Y | Y | Y | Y | Y | Y | Y | Y | Y | Y | Y | Y | Y | Y | Y | Y | Y | Y | Y |
|  | Are there complete outcome data? |  |  |  |  |  | Y | Y | Y | Y | Y | Y | Y | Y | Y | Y | Y | Y | Y | Y | Y | Y | Y | Y | Y | Y | Y |
|  | Are the confounders accounted for in the design and analysis? |  |  |  |  |  | N | N | N | Y | N | N | N | N | N | N | N | Y | Y | N | N | N | N | N | N | N | N |
|  | During the study period, is the intervention administered (or exposure occurred) as intended? |  |  |  |  |  | Y | Y | Y | Y | Y | Y | Y | Y | Y | Y | Y | Y | Y | Y | Y | Y | Y | Y | Y | Y | Y |
|  | Total score | 4 | 5 | 4 | 5 | 5 | 4 | 4 | 4 | 5 | 3 | 4 | 4 | 3 | 4 | 4 | 3 | 5 | 5 | 3 | 4 | 4 | 4 | 3 | 3 | 4 | 4 |

|  | | **QUANTITATIVE DESCRIPTIVE STUDIES** | | | | | | | | | | | | | | | | | | | | | | | | | | | | | |
| --- | --- | --- | --- | --- | --- | --- | --- | --- | --- | --- | --- | --- | --- | --- | --- | --- | --- | --- | --- | --- | --- | --- | --- | --- | --- | --- | --- | --- | --- | --- | --- |
|  | | Ailabouni et al., 2017 | Al-Jumaili & Doucette 2018 | Alldred et al., 2011 | Barker et al., 1982 | Barker et al., 2002 | Campagna et al., 2021 | Chen et al., 2018 | Deshmukh & Sommerville, 1996 | Dube et al., 2018 | Elliott, Lee, & Hussainy | Farner & Hicks 1976 | Fuller et al., 2022 | Garratt et al., 2020a | Garratt et al., 2020b | Gilmartin-Thomas et al., 2017 | Greene et al., 2005 | Hamrick et al., 2007 | Holmqvist et al., 2018 | Hughes et al., 2012 | Hughes, Wright, & Lapane 2006 | Jani et al., 2022 | Kaasalainen et al., 2010 | Karttunen et al., 2020 | Kirkevold & Engedal, 2009a | Kirkevold & Engedal, 2009b | Kirkevold & Engedal, 2010 | Lane et al., 2014 | Lee et al., 2015 | McCloskey et al., 2015 | McGillicuddy et al., 2016 |
| **GENERAL QUESTIONS** | Are there clear research questions? | Y | Y | Y | Y | Y | Y | Y | Y | Y | Y | Y | Y | Y | Y | Y | Y | Y | Y | Y | Y | Y | Y | Y | Y | Y | Y | Y | Y | Y | Y |
|  | Do the collected data allow to address the research questions? | Y | N | Y | Y | Y | Y | Y | Y | Y | Y | Y | Y | Y | Y | Y | Y | Y | Y | Y | Y | Y | Y | Y | Y | Y | Y | Y | Y | Y | Y |
| **QUANTITATIVE DESCRIPTIVE QUESTIONS** | Is the sampling strategy relevant to address the research question? | Y | Y | Y | Y | Y | Y | Y | Y | Y | Y | Y | Y | Y | Y | Y | Y | Y | Y | Y | Y | Y | Y | Y | Y | Y | Y | Y | Y | Y | Y |
|  | Is the sample representative of the target population? | Y | Y | Y | Y | Y | Y | Y | N | Y | Y | Y | Y | Y | Y | Y | Y | Y | Y | N | Y | N | Y | Y | Y | Y | Y | Y | N | Y | N |
|  | Are the measurements appropriate? | Y | Y | Y | Y | Y | Y | Y | Y | Y | Y | Y | Y | Y | Y | Y | Y | Y | Y | Y | Y | Y | Y | Y | Y | Y | Y | Y | Y | Y | Y |
|  | Is the risk of nonresponse bias low? | N | Y | Y | Y | Y | Y | Y | Y | Y | Y | Y | Y | Y | Y | Y | Y | Y | Y | Y | Y | Y | Y | N | Y | Y | Y | Y | Y | Y | Y |
|  | Is the statistical analysis appropriate to answer the research question? | Y | Y | Y | Y | Y | Y | Y | Y | Y | Y | Y | Y | Y | Y | Y | Y | Y | Y | Y | Y | Y | Y | Y | Y | Y | Y | Y | Y | Y | Y |
|  | Total score | 4 | 5 | 5 | 5 | 5 | 5 | 5 | 4 | 5 | 5 | 5 | 5 | 5 | 5 | 5 | 5 | 5 | 5 | 4 | 5 | 4 | 5 | 4 | 5 | 5 | 5 | 5 | 4 | 5 | 4 |

|  | | **QUANTITATIVE DESCRIPTIVE STUDIES** | | | | | | | | | | | | | | | | | | | | | | | | | | | | |
| --- | --- | --- | --- | --- | --- | --- | --- | --- | --- | --- | --- | --- | --- | --- | --- | --- | --- | --- | --- | --- | --- | --- | --- | --- | --- | --- | --- | --- | --- | --- |
|  | | Mercovich, Kyle, Naunton 2014 | Mitty 2009 | Nicholson & Damons, 2022a | Nicholson & Damons, 2022b | Paradiso et al., 2002 | Picton et al., 2021 | Pierson et al., 2007 | Prasanna et al., 2016 | Qian, Yu, & Hailey 2015 | Qian, Yu, & Hailey 2016a | Qian, Yu, & Hailey 2016b | Raban et al., 2020 | Roberts et al., 1998 | Santos et al., 2016 | Scott-Cawiezell et al., 2007 | Sefidani Forough et al., 2020 | Sefidani Forough et al., 2021 | Seifert & Johnston 2005 | Sharma et al., 2021 | Solberg et al., 2021 | Stasinopoulos et al., 2018 | Stokes, Purdie, & Roberts 2004 | Szczepura, Wild, & Nelson 2011 | Tangiisuran et al., 2018 | van den Bemt et al., 2009 | Vander Stichele et al., 1992 | Verrue et al., 2011 | Wright 2002 | Zimmerman et al., 2011 |
| **GENERAL QUESTIONS** | Are there clear research questions? | Y | Y | Y | Y | Y | Y | Y | Y | Y | Y | Y | Y | N | Y | N | Y | Y | Y | Y | Y | Y | Y | Y | Y | Y | Y | Y | Y | Y |
|  | Do the collected data allow to address the research questions? | Y | Y | Y | Y | Y | Y | Y | Y | Y | Y | Y | Y | Y | Y | Y | Y | Y | Y | Y | Y | Y | Y | Y | Y | Y | Y | Y | Y | Y |
| **QUANTITATIVE DESCRIPTIVE QUESTIONS** | Is the sampling strategy relevant to address the research question? | Y | Y | Y | Y | Y | Y | Y | Y | Y | Y | Y | Y | Y | Y | Y | Y | Y | Y | Y | Y | Y | Y | Y | Y | Y | Y | Y | Y | Y |
|  | Is the sample representative of the target population? | N | Y | Y | Y | Y | Y | Y | Y | N | N | N | Y | Y | Y | Y | N | Y | N | Y | Y | Y | Y | Y | N | Y | Y | Y | Y | Y |
|  | Are the measurements appropriate? | Y | Y | Y | Y | Y | Y | Y | Y | Y | Y | Y | Y | Y | Y | Y | Y | Y | Y | Y | Y | Y | Y | Y | Y | N | Y | Y | Y | Y |
|  | Is the risk of nonresponse bias low? | Y | N | Y | Y | Y | Y | Y | Y | Y | Y | Y | Y | Y | Y | Y | Y | Y | N | Y | Y | Y | Y | Y | Y | Y | Y | N | Y | Y |
|  | Is the statistical analysis appropriate to answer the research question? | Y | Y | Y | Y | Y | Y | Y | Y | Y | Y | Y | Y | Y | Y | Y | Y | Y | Y | Y | Y | Y | Y | Y | Y | Y | Y | Y | Y | Y |
|  | Total score | 4 | 4 | 5 | 5 | 5 | 5 | 5 | 5 | 4 | 4 | 4 | 5 | 5 | 5 | 5 | 4 | 5 | 3 | 5 | 5 | 5 | 5 | 5 | 4 | 4 | 5 | 4 | 5 | 5 |

|  | | **MIXED-METHODS STUDIES** | | | | | | | | | |
| --- | --- | --- | --- | --- | --- | --- | --- | --- | --- | --- | --- |
|  | | Barber et al., 2009 | Breen, Williams, & Wroth., 2023 | Dilles et al., 2011 | Hilleary & Ferrini 2011 | Kemp, Luo, & Ball., 2012 | Lim et al 2016 | Mahmood, Chaudhury, & Gaumont., 2012 | Scott-Cawiezell et al., 2009 | Sikma et al., 2014 | Vermeulen et al., 2017 |
| **GENERAL QUESTIONS** | Are there clear research questions? | Y | Y | Y | N | Y | Y | Y | Y | Y | Y |
|  | Do the collected data allow to address the research questions? | Y | Y | Y | Y | Y | Y | Y | Y | Y | Y |
| **MIXED-METHODS STUDIES QUESTIONS** | Is there an adequate rationale for using a mixed methods design to address the research question? | Y | Y | Y | Y | Y | Y | Y | Y | Y | Y |
|  | Are the different components of the study effectively integrated to answer the research question? | Y | Y | Y | N | Y | Y | Y | Y | Y | Y |
|  | Are the outputs of the integration of qualitative and quantitative components adequately interpreted? | Y | Y | Y | N | Y | Y | Y | Y | Y | Y |
|  | Are divergences and inconsistencies between quantitative and qualitative results adequately addressed? | Y | Y | Y | Y | Y | N | Y | Y | Y | Y |
|  | Do the different components of the study adhere to the quality criteria of each tradition of the methods involved? | Y | Y | N | N | Y | Y | Y | Y | Y | Y |
|  | Total score | 5 | 5 | 4 | 2 | 5 | 4 | 5 | 5 | 5 | 5 |
