# Supplementary material for: Medication administration in aged care facilities: A mixed‐methods systematic review
Source: J Adv Nurs. 2024 Jul 7;81(2):621–40. doi: 10.1111/jan.16318 (PMC11729541; doi:10.1111/jan.16318)
Supplement: Supplementary file 3 — Table S2. [file JAN-81-621-s002.docx]

**Non-randomized studies (N=21)**

| **Reference** (Country) | **Methodology** | | | ***Participants***  *(N, age, % female)* | **Study focus** | **Key findings** |
| --- | --- | --- | --- | --- | --- | --- |
|  | Design | Setting | Method |  |  |  |
| Alenius & Graf 2016  (Sweden) | Non-randomized study | 2 Residential Aged Care Facilities | Purposive sample  Surveys  *Baseline:* 55% response rate in control group, 56% response in intervention group.  *Follow-up*: 42% response rate in control group, 55% response rate in intervention group  Chi-square test, Student t-test | *Baseline*  Control: 29 Nurses Aides/Assistant Nurses  Age = not stated  Sex = not stated  Intervention: 37 Nurses Aides/Assistant Nurses  Age = not stated  Sex = not stated  *Follow-up*  Control: 23 Nurses Aides/ Assistant Nurses  Age = not stated  Sex = not stated  Intervention: 36 Nurses Aides/Assistant Nurses  Age = not stated  Sex = not stated | Medication error, Technology | Staff at the intervention site less concerned about medication administration errors than those at the control site. Significant reduction in perceived risk of error (*p*<0.001), improved perception of the medication administration process (*p*=0.012). |
| Badawoud et al., 2018  (USA) | Non-randomized study | 6 Residential Aged Care Facilities | Convenience sample  Surveys (response rate 78.6%), Intervention (personalised approach to medication administration)  Descriptive analysis, Pearson exact chi-square test, Tabulation of qualitative responses | 22 Medication administration staff  Age=not stated  Sex=not stated  *Resident directed medication administration*: 51 Residents  Age (Mean)= 86 years (sd. 0.6)  Sex = 88% female  *Facility directed medication administration*: 81 residents  Age (Mean)= 83 years (sd. 4.2)  Sex=75% female | Medication error, Time | Resident-directed medication administration sites experienced fewer medication passes and errors than facility-directed administration sites, and spent less time giving medications. Resident satisfaction about medication administration process was perceived as a 'yes' by the majority across both sites (64-75% residents, 100% of family members). |
| Baril et al., 2014  (Canada) | Quasi-experimental study | 6 Residential Aged Care Facilities | Convenience sample  Audit of voluntary reporting of errors 2008-2012  Descriptive analysis, two-sample t-test | 800 Residents  Age = not stated  Sex = not stated  3,087 Medication error records | Medication error, Technology | 80% of medication errors sat within four categories: medication not administered, dose/flow error, wrong patient, or 'other'. Medication distribution technology significantly increased reporting of medication errors (*t*-=-7.46, *df*=36, *p*=.000) and decreased accidents (*p*=.000). |
| **Reference** (Country) | **Methodology** | | | ***Participants***  *(N, age, % female)* | **Study focus** | **Key findings** |
|  | Design | Setting | Method |  |  |  |
| Carvajal et al., 2016  (Spain) | Non-randomized study | 10 Residential Aged Care Facilities | Purposive sample  Audit of records, Observation  Descriptive analysis, Student t-test, Chi-square test, Mann-Whitney U test, McNemar test. | 1875 Residents  Age (Mean)= 85.7 years (sd. 7.8)  Sex=71.9% female | Dose form modification | 33% experienced crushing of medication, more frequently for females (*p=0*.012). Common reasons for crushing/dose form alteration were ‘behaviour disorder’ (53.5%), swallowing issues (32.4%). Most common suggestion through intervention was dose form changes (78.6%, but only 40.6% acceptance 30 days post recommendation). |
| Chen et al., 2020  (Australia) | Non-randomized study | 3 Residential Aged Care Facilities | Purposive sample  WOMBAT software for observations  Descriptive analysis, Mann-Whitney U test, ANOVA. | 24 Enrolled nurses  Age=not stated  Sex=not stated  6 Registered Nurses  Age=not stated  Sex=not stated | Dose form modification, Time | Average of 5 minutes per resident per medication round. 15% of medication crushed in regular units; memory units required an additional minute, and 42% of medications were crushed. Average amount of time for 6 scheduled medication rounds was 5.3 hours/day; breakfast rounds took the longest (average of 1.92 (sd. 0.53) hours spent with a median of 22 residents). |
| Deshmukh & Sommerville 1996  (UK) | Non-randomized study | 2 Residential Aged Care Facilities | Convenience sample  Audit of policies, Intervention (medication reviews)  Descriptive analysis, Chi-square test, paired t-tests. | 60 Residents  Age (Mean) = 84 years (sd. 9)  Sex =87% female | Medication error, Medication omission | 173 recommendations and interventions, 52% implemented. Proportion of medication given correctly increased from 67.1% to 79.6% for regular medications at one site (*p*<0.05). Omissions decreased from 9.4 to 3.6% (*p*<0.05). Mean number of medications reduced from 7.14 (sd.3.39) to 5.68 (sd.2.90). |
| Eide & Schjott 2001  (Netherlands) | Non-randomized study | 7 Residential Aged Care Facilities | Convenience sample  Surveys  Chi-square test, Students t-test | *Intervention:* 5 sites; 187 Residents  Age (Mean)=85.7 years  Sex=75% female  *Control:* 2 sites; 79 Residents  Age (Mean)=87.2 years  Sex= 85% female | Time | Residents in the intervention group were less likely to have hypnotics administered before 9pm (compared to previous sample pre-intervention in 1995). Reduction was from 40% pre-9pm in 1995 to 13% in 2000. In the control group, 62.9% of hypnotics were given pre-9pm in 2000. |
| Elliott et al., 2020  (Australia) | Non-randomized study | 52 Residential Aged Care Facilities | Systematic random sample  Semi-structured interviews  Descriptive analysis, two-tailed t-test, Thematic approach | 73 Staff  Age=not stated  Sex=not stated  73 Residents  Age (Median)= 85 years  Sex=54.8% female | Medication administration challenges, Technology (Electronic medication records) | 97.3% of participants had an interim medication administration chart when discharged to aged care, only 2.7% experienced missed/delayed doses. These charts were helpful and effective, allowed medication administration without waiting for the GP. Electronic charts required an update from the pharmacy/GP, required a locum to avoid omissions and errors. |
| Fei, Robinson, & MacNeil 2019  (Canada) | Case study | 3 Residential Aged Care Facilities | Convenience sample  Observation, Survey (response rate not stated)  Descriptive analysis, Time analysis | 554 Residents  Age = not stated  Sex = not stated  74 Nurses  Age = not stated  Sex = not stated  60 Health Care Workers  Age = not stated  Sex = not stated | Technology (Electronic medication records) | Implementation of electronic medication administration record led to 35.1% overall decrease in incidents, omitted doses (39%), wrong doses (40%). Decrease in average round time of 32mins. Staff surveyed found the change positive, it increased efficiency and safety, gave extra time to engage with residents and family, decreased errors. |
| **Reference** (Country) | **Methodology** | | | ***Participants***  *(N, age, % female)* | **Study focus** | **Key findings** |
|  | Design | Setting | Method |  |  |  |
| Lau et al., 2003  (Hong Kong) | Before-after study | 85 Residential Aged Care Facilities | Random sampling, Voluntary participation  Audit, Observation, Evaluation  Descriptive analysis, McNemar’s test. | 85 Residential Aged Care Facilities | Education, Medication error | Proportion of sites with medication administration issues decreased by 27% (*p*<0.05) after the interventions had taken place. Pre-intervention 69% of the sites did not give medication according to the dispensing label, reduced to approximately 20% post-intervention. |
| McDerby et al., 2019  (Australia) | Pilot controlled trial | 2 Residential Aged Care Facilities | Purposive sample  Observational audits, Intervention (onsite pharmacist), Document review  Descriptive analysis, Fishers exact test, paired and independent t-tests. | *Control:* 43 Residents  Age (Median) = 87 years (IQR 82-91)  Sex = 58% female  *Intervention:* 74 Residents  Age (Median) = 87 years (IQR 82-91)  Sex =70.3% female | Dose form modification, Time | Inappropriate dose form modification proportion reduced significantly from 24% to 0%; mean time spent on medication rounds per resident decreased (4.8 (sd. 1.1) to 3.2 (sd. 1.7), *p*<0.05) - average potential time saving of 61 minutes per medication round. Most common documented incident was medication omission (115 reports). Rate of incident reported per resident increased from 0.31 to 0.42 at follow up. |
| Park et al., 2013  (South Korea) | Non-equivalent comparison group study | 1 Residential Aged Care Facility | Purposive sample  Interviews, review of medical records,  Descriptive analysis, ANOVA, t-tests or Chi-square. | *Baseline*  *Comparison:* 24 residents  Age (Mean)= 77 years (sd.7.2)  Sex = 75% female  *Intervention:* 23 Residents  Age (Mean)= 78 years (sd.7.0)  Sex = 70% female | Medication adherence | At baseline: intervention group had a mean of 3.1 medication administration times per day (sd. 1.6), comparison group had a mean of 2.7 (sd. 1.0). Only 34% self-administered. No significant difference between groups for medication adherence from baseline to follow-up (x^2^=0.21, *p*=0.88). Significant improvement in self-care behaviour in intervention group, decrease in the comparison group (mean difference 7.1 points, *p*<0.005). |
| Sanchez et al., 2021  (France) | Non-randomized pre-post study | 519 Residential Aged Care Facilities | Convenience sample  Retrospective review of medication records  Descriptive analysis, Difference-in-differences multilevel Poisson regression | *Comparison*: 66,463 residents  Age = not stated  Sex = not stated  *Intervention:* 29,780 residents  Age = not stated  Sex = not stated | Time | Average daily use of medication decreased in both groups (RR=0.98, 95%CI 098-0.99), intervention did not have a significant effect (exponentiated DID μ 1.00, 95%CI 0.99-102, *p*=0.45). Main outcome: significantly lower use of meds RR=0.98 (95%CI, 0.97-0.99). Secondary outcomes: total number of different drugs prescribed per resident (decrease in use OR=-/95, 95%CI 0.95-0.97, *p*<0.0001), use of anti-hypertensive and hypnotics (both decreased significantly, but no difference between groups). |
| Stuijt et al., 2013  (Netherlands) | Prospective before-after study | 6 Residential Aged Care Facilities (Psycho-geriatric wards) | Convenience sample  Observation  Descriptive analysis, Student t-test, Chi-square test, Mann-Whitney U test, Binary logistic regression. | *Baseline:* 60 Residents  Age (Mean) = 80 years (sd.8.5)  Sex = 73% female  *First evaluation:* 55 Residents  Age (Mean) = 80 years (sd.7.8)  Sex = 67% female  *Second evaluation:* 62 Residents  Age (Mean) = 82 years (sd.7.6)  Sex = 72.6% female | Dose form modification, Medication error | *Primary outcome:*  Overall proportion of medication administration errors. First evaluation, overall proportion of MAEs decreased significantly by 23.9% and crushing errors by 63.2% (*p*=0.935; *p*=0.005). By the second evaluation, proportion of crushing errors remained significantly lower (52.6% reduction, *p*=0.045), but when adjusted for confounders, the odds of observing this type of error during second evaluation was non-significant (OR=0.71, 9%CI, 0.28-1.80). |
| **Reference** (Country) | **Methodology** | | | ***Participants***  *(N, age, % female)* | **Study focus** | **Key findings** |
|  | Design | Setting | Method |  |  |  |
| Tenhunen, Tanner, & Dahlen 2014  (USA) | Single-group descriptive study with pre-test/post-test design | 2 Residential Aged Care Facilities | Convenience sample  Pre- and post-test (58% participation rate)  Descriptive analysis, Paired t-tests | 72 Licenced Nursing Staff  Age = not stated  Sex = not stated | Education | Significant increase in knowledge at only one site (site 1: *p*=0.04). Average score at pre-test for site 1 was 23.66/36 (sd. 4.25), at site 2 22.31/36 (sd. 3.07). Post-test site 1 average 25.09 (sd. 3.06) and site 2 averaged 24.78 (sd. 3.23) points. |
| van Welie et al., 2016  (Netherlands) | Prospective uncontrolled intervention study | 3 Residential Aged Care Facilities | Sample method unspecified  Intervention, Disguised observation  Descriptive analysis, Chi-square test, Students t-test | 36 Nurses/Nursing assistants  Age = not stated  Sex = 92%  197 Residents  Age (Mean) = 82 years  Sex = 63% female | Dose form modification | *Primary outcome measure:* The crushing error rate decreased from 3.1% to 0.5%, RR=0.15 (95%CI 0.05-0.51). Reduction in crushing errors related to residents with swallowing difficulties reduced from 87.5% (21 errors to 24 medications) to 30% (3 errors per 10 medications) (RR 034, 9%CI 0.13-0.89). |
| Verrue et al., 2010  (Belgium) | Before-after study | 2 Residential Aged Care Facilities | Convenience sample  Educational intervention, observation  Descriptive analysis, Chi-square test | *Site 1:*  72 Residents  Age (Mean) = 86 years (sd.7.1)  Sex = 81%  *Site 2:*  27 Residents  Age (Mean) = 86 years (sd.5.3)  Sex = 67% female  *Training sessions:* 69 staff  Age = not stated  Sex = not stated | Education, Medication error | Average number of medications was similar pre/post- between both sites (Site 1, 8.1 medications (sd. 3.5) to 8.2 (sd. 3.5); Site 2, 8.2 medications (sd. 3.9) to 7.9 (sd. 3.0)). Overall error rate significantly decreased, (*p*<.001 (site 1); *p*=.049 (site 2)). Post-intervention error rate reduced from 6.3% to 2.0% and 3.2%R to 1.7%. Post-intervention, most common scored error was omission (62.2%, 27.3%). Education and information about crushing tablets was appreciated by 88.4% of staff. |
| Vogelsmeier et al., 2022  (USA) | Controlled pilot study | 1 Residential Aged Care Facilities | Sampling method not stated  Naïve observation, Field notes  Descriptive analysis, z-test | 10 Staff  Age (Mean) = 38 years (sd.12.2)  Sex = 80% female  70 Residents  Age (Mean) = 81 years (sd.10.7)  Sex = 74% female | Medication error, Technology | 12% of administrations were errors. Significantly less errors in the intervention unit: wrong dose (*p* <0.01), wrong medication (*p* <0.01), wrong route (*p* <0.01). Time errors higher in intervention unit, blamed on late starts or interruptions, versus memory or nursing judgement. Staff would document that it was given/resident refused rather than omission decision (omissions 4% of errors (intervention) versus 34% of errors (control) (*p*=0.03). |
| Wagner, Wahlberg, & Worning 1994  (Denmark) | Longitudinal study | 1 Residential Aged Care Facility | Convenience sample  Surveys  Descriptive analysis, Wilcoxon’s two-sample location test for signed rank sum test. | *Baseline:* 42 Residents  Age (Mean) = 83 years  Sex = 60% female  *Follow up 1:* 48 Residents  Age= not stated  Sex = 63% female  *Follow up 2:* 39 Residents  Age= not stated  Sex = 69% female  *Follow up 3:* 35 Residents  Age= not stated  Sex = 69% female | Self-administration | Intervention promoted self-administration of medication where possible. By the second survey, 40% of residents were self-administering and 52% had help from staff but kept their own medication in their rooms and were actively engaged in the process. No hospitalisations, incorrect dosage incidents occurred. Total number of regularly administered medications per day reduced (*p*<0.05) to average of 3.6 medications per person (range 0-9). Time gained by staff was used to better educate and inform residents about medications. |
| **Reference** (Country) | **Methodology** | | | ***Participants***  *(N, age, % female)* | **Study focus** | **Key findings** |
|  | Design | Setting | Method |  |  |  |
| Ward et al., 2008  (USA) | Quasi-experimental study | 1 Residential Aged Care Facility | Convenience sample  Review of medication records  Descriptive analysis, t-tests, Chi-square tests. | 20 Discharged patients  Age (Mean) = 83 years (sd.9.2)  Sex = 55% female | Medication omission | Mean time delay between arrival to first dose was 12.5 hours (sd. 7.45), no significant difference in mean times between groups. Mean number of doses of different medications omitted was 3.4 (2.60) per patient; 91% of all omitted doses involved only one dose of a singular medication. Intervention group had larger number of omitted doses of medication (*p*<0.05) and higher number of omitted doses that had high/medium potential for harm (*p*<0.05). |
| Wild, Szczepura, & Nelson 2011  (UK) | Pre-post intervention study | 13 Residential Aged Care Facilities | Convenience sample  Survey questionnaire (response rate 80%), Interviews, Focus groups  Descriptive analysis, Ranks Mann-Whitney test, Content analysis | Pre-intervention: 49 staff  35 Care staff, 14 Registered Nurses  Age = not stated  Sex = not stated  Post-intervention: 39 staff  27 Care staff, 12 Registered Nurses  Age = not stated  Sex = not stated | Medication error, Technology | Before the barcode system, 40% of staff in residential care noted that near misses occurred, but nurses in the nursing home reported none. Significant difference between near misses, staff groups and type of site pre-barcode system (Mann-Whitney, *z*=-2.74, *p*<0.05). Some near misses were justified by nurses as legitimate deviations (using professional judgement). Most common reason for error pre/post was interruptions (100% of carers pre, 79% post; 86% of nurses pre, 42% post). |

**Randomised Control Trials (N=5)**

| **Reference** (Country) | **Methodology** | | ***Participants***  *(N, age, % female)* | **Study focus** | **Key findings** |
| --- | --- | --- | --- | --- | --- |
|  | Setting | Method |  |  |  |
| Dugre et al., 2021  (Australia) | 8 Residential Aged Care Facilities | Cluster-randomized controlled trial (Open label, matched paired cluster)  Random sample  Descriptive analysis, Negative binomial regression | *Baseline*  *Comparison:* 143 Residents  Age (Median) = 88 years (IQR 80-92)  Sex = 78% female  Lost to follow-up = 30 residents  *Intervention*: 99 Residents  Age (Median) = 86 years (IQR 80-92)  Sex = 67% female  Lost to follow-up = 49 residents | Medication error | Mean medication incident rates 95 (intervention) and 66 (comparison) per 100 patient years over 12-month period (Adj IRR 1.13, 95%CI 0.53-2.38). 12-month pre/post incident rate halved among participants in the intervention group (Adj IRR 0.56, 95%CI 0.38-0.08). The most common incident classification was administration error (88.8% in intervention group; 81.5% in comparison group). Omission of medication was 26.4% in intervention group; 23.7% in the comparison group. |
| Forman et al., 2021  (USA) | 1 Residential Aged Care Facility | Exploratory, single-centre, open-label, randomized, crossover study  Random sample  Rating scales  Descriptive analysis, Safety analysis | 18 Residents  Age (Mean)= 65 years (sd. 4.9)  Sex = 50% female | Resident involvement | Residents found chocolate pudding, vanilla ensure most palatable. Swallowability was rated similarly for all four options. 88% of participants were able to open the medication capsules and empty the contents on each day. |
| Kolcu & Ergun 2020  (Turkey) | 2 Residential Aged Care Facilities | Randomized Control Trial  Purposive sampling, random assignment  Intervention, questionnaires  Chi-square test, Wilcoxon signed-rank test, Mann-Witney U-test. | *Control:* 37 Residents  Age= 43.2% between 65-74 years  Sex = 43.2% female  *Intervention:* 37 Residents  Age= 48.6% between 65-74 years  Sex = 48.6% female | Medication adherence | *Primary outcome:* Post-test medication adherence significantly higher in the intervention group (x^2^=15.77, *p*<0.001). Interventions involved adherence follow-up, health education, medication boxes distributed. |
| Sluggett et al., 2020a; 2020b  (Australia) | 8 Residential Aged Care Facilities | Nonblinded, matched-pair, cluster Randomized controlled trial  Random sample  Chart review (baseline data), Surveys, Application of validated 5-item tool, review of risk management and reporting software  Constrained longitudinal data analysis | *Comparison:* 143 Residents  Age (Mean)= 86 years (sd8.3)  Sex = 78% female  *Intervention:* 99 Residents  Age (Mean)= 86 years (sd7.8)  Sex = 68% female | Time | *Primary outcome:* mean number of administration times in the intervention group was reduced at follow up (-0.36, 95% CI -0.63 to -0.09, *p* =0.01). Mean number of administration times at baseline similar between both arms (3.9 (sd.1.5); 4.0 (sd.1.8)) with ICC (baseline) of 0.003). Post intervention, significant difference in number of administration times for regular medication between groups (mean difference-0.36, 95%CI -0.63, -0.09, *p*=0.01). 8 months follow-up, significant reduction in mean number of daily medication administration times for intervention group (-0.38, 95%CI -0.69 to -0.07, *p=0.*014), and at 12 months follow up (-0.47, 95%CI -0.84 to -0.09, *p*=0.014). |

**Quantitative Descriptive Studies (N=59)**

| **Reference** (Country) | **Methodology** | | | ***Participants***  *(N, age, % female)* | **Study focus** | **Key findings** |
| --- | --- | --- | --- | --- | --- | --- |
|  | Design | Setting | Method |  |  |  |
| Ailabouni et al., 2017  (New Zealand) | Cross-sectional study | 307 Residential Aged Care Facilities | Random sample  National Survey (29.6% response rate)  Descriptive Analysis | 91 Registered Nurses  Age = not stated  Sex = not stated | Medication administration challenges, Time | Concerns included medication reconciliation, new admissions, access to prescribers, disruptions during rounds, and information sharing. Challenges included length of time of rounds, resident refusals. |
| Al-Jumaili & Doucette 2018  (USA) | Cross-sectional study | 11 Residential Aged Care Facilities | Purposive sample  Medication chart review; Surveys (100% response rate)  Descriptive analysis, Binary logistic regression | 11 Directors of Nursing  Age = not stated  Sex = not stated  11 Registered Nurses  Age = not stated  Sex = not stated  755 Residents  Age = not stated  Sex = not stated | Medication error | Incidence of Adverse Drug Events of 6.13 per 100 residents per month. Significant positive associations: Dementia diagnosis, psychotropic medication, opioids, warfarin, heavy nurse workload (*p*<.05). Sites with more collaboration between nursing staff and physicians significantly less likely to have adverse events (OR=.78). |
| Alldred et al., 2011  (UK) | Cross-sectional study | 55 Residential Aged Care Facilities | Purposive sample  Review of error observation forms  Descriptive analysis, t-test, Logistic regression. | 233 Resident medication records | Medication error | Mean number of errors per resident, 0.17-0.20. Odds of administration error for tablets/capsules not in a monitored dosage system were double those that were (Adj OR 2.14, 95% CI 1.02-4.51, *p*=.04) |
| Barker et al., 2002  (USA) | Prospective cohort study | 12 Skilled Nursing Facilities (and 24 hospital sites) | Stratified random sample  Observation  Descriptive analysis, Tukey test | 1,451 Doses of medication | Medication error | Average error rate across sites was 19.5%, when ‘wrong time’ errors excluded, average error rate was 11.3%. No statistically significant difference in error rates between site types (*p*=.82) or site size (*p*=.39). |
| Barker et al., 1982  (USA) | Cross-sectional study | 62 Residential Aged Care Facilities | Random sample  Observation  Randomised block analysis of variance | 3,051 Opportunities for error | Medication error | Most common form of error was omission (41.5%), with a total estimated error rate of 12.2% of all doses across sites (sd. 2.95). |
| Campagna et al., 2021  (Italy) | Cross-sectional study | 50 Residential Aged Care Facilities | Convenience sample  Observation  Descriptive analysis | 175 Residents  Age (Mean) = 83.1 years (sd. 11)  Sex =71.4% female | Medication omission | Delayed drug administration most frequent missed nursing care type (34.5%). Half of missed nursing care attributed to inadequate staffing, 62.1% of occurred in the morning. Common unavoidable factor: clinical events impacting working routine (51.5%). |
| Chen et al., 2018  (Australia) | Cross-sectional study | 10 Residential Aged Care Facilities | Purposive sample  Expert panel, Validation and application of simplification tool  Modified nominal group technique, Inter-rater reliability analysis | 50 Residents  Age (Mean) = 82 years (sd. 9.8)  Sex =76% female | Time | The most common recommendation using the tool was to change a dose administration time (75%) as part of the simplification process. |
| **Reference** (Country) | **Methodology** | | | ***Participants***  *(N, age, % female)* | **Study focus** | **Key findings** |
|  | Design | Setting | Method |  |  |  |
| Deshmukh & Sommerville 1996  (UK) | Cross-sectional study | 2 Residential Aged Care Facilities | Convenience sample  Medication chart audit, Surveys  Descriptive analysis | 79 residents  Age (Mean) = 84 years (sd. 9)  Sex =80% female | Medication error, Resident involvement | 58% compliance with medication administration guidance. Most common error was not properly identifying residents, and not observing full dose ingested. 0.5% of medications refused. Four residents wanted to self-administer, but deemed non-competent. |
| Dube et al., 2018  (Canada) | Cross-sectional study | Residential Aged Care Facilities (number not specified) | Purposive sample  Retrospective audit  Descriptive analysis, z-tests | 494 Residents  Age = 46.9% between 65-84 years, 53% over 85 years  Sex =70.2% female | Medication error | Average provincial call rate for "wrong medication" to the wrong resident was 2.25 calls per resident per 1000 people living in nursing homes. 45% of this error type occurred during evening shifts. |
| Elliott, Lee, & Hussainy  2016  (Australia) | Cross-sectional study | 1 Residential Aged Care Facility | Convenience sample  Retrospective audit of medication orders  Descriptive analysis | 88 Residents  Age (Mean) = 86 years (sd. 6.9)  Sex = 70.5% female | Medication error, Technology | There were 125 discrepancies between paper orders and the electronic charts, 50% due to pharmacy entry error. 24% of residents experienced an administration error as a result. Most common error was omission (44%). |
| Farner & Hicks 1976  (USA) | Cross-sectional study | 3 Residential Aged Care Facilities | Purposive sample, Work sampling  Observation  Descriptive analysis, ANOVA, One tailed t-test | 50-53 Residents per site | Dose administration aids, Time | Drug distribution systems influenced the amount of time spent doing medication administration. Potential time savings of 218 (24-unit dose system) and 359 hours (72-unit dose system) per month. |
| Fuller et al., 2022  (Canada) | Quantitative descriptive study | 1 Residential Aged Care Facility | Purposive sample  Audit and document review of incident reports  Descriptive analysis, Student t-tests, ANOVA, Q-Q plots, Chi-square tests, Fisher’s exact test, Content analysis | 270 Medication incident reports (June 2015-Oct 2017) | Medication error, Technology | 71.9% of medication incidents occurred at the administration level, 2.3% resulted in temporary harm. Omission (43.7%) and wrong time (22.6%) were the most common error. Workarounds included documenting process prior to actually giving the medication (44.9%). Refusals were considered self-administration issues and recorded as omissions. |
| Garratt et al., 2020a, 2020b  (New Zealand) | Cross-sectional study | 374 Residential Aged Care Facilities | Purposive sample  Retrospective audit of medication records  Descriptive analysis, Chi-square tests, ANOVA, independent samples t-tests, Kruskal Wallis H test, Ranks Mann-Whitney test. | 11,015 residents  Age (Median) = 87 years  Sex = 68% female | Medication omission | Mean rate of 3.59 doses omitted per 100 dispensed doses per resident. 73% of residents had at least one dose omission during the timeframe. Most common omission type was 'not administered' (49.9%) followed by refused (34.6%). 48% of dose omissions lacked an explanation why, pain relief and laxatives most administered and omitted medications. |
| Gilmartin-Thomas et al., 2017  (UK) | Prospective cohort study | 10 Residential Aged Care Facilities | Purposive sample  Observation  Descriptive analysis, Poisson regression | 41 Staff  Age= not stated  Sex=85% female  823 Residents  Age (Mean)=82 years  Sex=62% female | Dose administration aids, Medication error | Higher error rate for original packaging versus multi-compartment compliance aids (9.3% versus 3.1%, RR = 2.3, 95%CI 1.1-4.9, *p*=.03). 7.1% administration error rate. Median of 65mins (range 8-245mins) per medication round. Lower error rate for multiple doses compared to singular medication dose (RR=0.643, 95%CI 0.41-0.97, *p*=0.043). Wrong time (2.9% error rate), omission (1.6% error rate) and wrong dose (1.2% error rate). |
| **Reference** (Country) | **Methodology** | | | ***Participants***  *(N, age, % female)* | **Study focus** | **Key findings** |
|  | Design | Setting | Method |  |  |  |
| Greene et al., 2005  (USA) | Cross-sectional study | 385 Residential Aged Care Facilities | Convenience sample  Survey (100% response rate), Retrospective review  Descriptive analysis | 10,920 Medication error records. | Medication error | Over 9 months only three sites reported zero errors. Mean number of errors was 28.4 errors across all sites, median number was 15.0 errors. Most common types of error: dose omission (49.7%), wrong dosage (22.6%). Primary causes of error were human factors (53.3%) and communication (40.2%). |
| Hamrick et al., 2007  (USA) | Time-motion study | 2 Residential Aged Care Facilities | Convenience sample  Observation  Cost-minimization analysis, Time-motion analysis | 10 Registered Nurses  Age=not stated  Sex=not stated | Time | An additional dose of oral medication for one patient during an administration round with other medications took 45 seconds, but a new medication administration round time (external to other charted doses) added 63 seconds. |
| Holmqvist et al., 2018  (Sweden) | Time-motion study | Residential Aged Care Facilities, Private homes | Purposive sample  Observations (WOMBAT software)  Descriptive analysis, Monte-Carlo test | 27 Registered Nurses  Age=not stated  Sex=93% female | Medication administration challenges | 23% of nurses time spent on medication management. Rate of 1.2 interruptions per hour (95%CI 1.1-1.4), 30% during medication management. Documentation took up 4.2% of their observable time in nursing homes versus private homes (1.8%, *p*=.002). |
| Hughes, Wright, & Lapane 2006  (USA) | Cross-sectional study | 6, 344 Residential Aged Care Facilities | Purposive sample  Retrospective review  Descriptive analysis, Multiple logistic regression | 16,052 surveys from Online Survey and Certification of Automated Records data | Staffing | Larger sites more likely to use medication technicians (OR 1.66, 95% CI 1.19-2.30). Sites that used medication technicians were more likely to have medication error rates (10.1% versus 7.3%) than homes without medication technicians. |
| Hughes et al., 2012  (Northern Ireland, New Zealand) | Cross-sectional study | 70 Northern Ireland, 14 New Zealand Residential Aged Care Facilities | Stratified random sample  Survey (35.5% response rate for Northern Ireland managers, 10.1% for nurses; 90.9% for New Zealand managers, 71% for nurses)  Pre-specified scoring system, Chi-square test, t-test. | *Northern Ireland*  50 Managers; 142 Registered Nurses  Age=not stated  Sex=not stated  *New Zealand*  10 Managers; 44 Registered Nurses  Age=not stated  Sex=not stated | Staffing, Treatment culture | The average number of nurses per resident in Northern Ireland was 0.28, compared to 0.06 full time equivalent staff in New Zealand. Despite different funding and staffing structures, no major difference between mean treatment culture scores. |
| Jani et al., 2022  (UK) | Multi-centre, prospective, cross-sectional study | 1 Residential Aged Care Facility | Convenience sample  Real-life observer-reported outcomes: ClinSearch Acceptability Score Test  Accountability reference framework (Multivariate analysis) | 97 Observer reports | Medication acceptability, Self-administration | 58% of residents in care home had 10+ medications prescribed. ‘Acceptability’ defined as willingness and ability to self-administer or be administered. Medications were less accepted in the care home, 47% of the confidence ellipses within the 'negative accepted' zone. Patients without swallowing disorders in the care home reported as having negative observations more that those without. |
| **Reference** (Country) | **Methodology** | | | ***Participants***  *(N, age, % female)* | **Study focus** | **Key findings** |
|  | Design | Setting | Method |  |  |  |
| Kaasalainen et al., 2010  (Canada) | Cross-sectional study | 9 Residential Aged Care Facilities | Convenience sample  Survey (based on MAS-NAS scale) (response rate 59%)  Descriptive analysis, Independent two-sample t-tests, Content analysis | 165 Nursing staff  Age (Mean)=45.3 years (sd. 11.67)  Sex=92% female | Safety, Staffing | Significant difference between staff type satisfaction with the current system (Registered Nurses mean=6.60, sd. 2.42 vs. Registered Practice Nurses mean=7.37, sd. 1.93, *t*=2.38; *p*<.02). Safe administration was influenced by a range of factors: staff support, medication packaging, resource access, interruptions, education. Barriers: workload issues, time constraints, limited communication, staff competencies, and bulky medication carts. |
| Karttunen et al., 2020  (Finland) | Cross-sectional study | Residential Aged Care Facilities (number not specified) | Total sampling  Survey (response rate 39.4%)  Descriptive analysis, Chi-square tests, ANOVA, Kruskal-Wallis test, Mann-Whitney U-test. | 429 Nursing staff  Age (Mean)=46 years (sd. 11.09)  Sex=98% female | Dose form modification, Medication error | 44% deviated occasionally when administering medication. Older age groups more likely to deviate (*p*<.001). 33%-34% would crush sustained or enteric coated tablets against advice. More knowledge of pharmacology, infection control, or higher self-assessed medication calculation skills indicated better adherence to guidelines. |
| Kirkevold & Engedal 2009a  (Norway) | Cross-sectional study | Residential Aged Care Facilities across 54 municipalities | Convenience sample  Structured Interviews  Descriptive analysis, Multi-level model for regression analysis | 444 Residents (Dementia Unit)  Age=not stated  Sex=not stated  1057 Residents (Regular care)  Age=not stated  Sex=not stated | Dose form modification | 17% of residents in dementia units,11% of residents in regular units experienced covert administration. 95% of covert administration was routine, poorly documented. Over 50% explained as result of resident non-compliance, 28% swallowing issues. Dementia, low function in Activities of Daily Living, aggression explanatory factors. |
| Kirkevold & Engedal 2009b  (Norway) | Cross-sectional study | 65 Residential Aged Care Facilities | Purposive sample  Survey (response rate not stated)  Descriptive analysis, Multilevel multivariate logistic regression | 623 Residents (Dementia Unit)  Age (Mean)=84 years (sd. 8.6)  Sex=73.5% female  1320 Residents (Regular care)  Age (Mean)=86 years (sd. 8.9)  Sex=68.5% | Dose form modification | 23.5% of residents had medication covertly given in food/beverages, this decision was typically made by nursing staff in consultation with the physician or by the physician alone (61.4% in dementia units, 52.8% in regular units). Higher chance of antipsychotic use if medication covertly given (*p*<.001). |
| Kirkevold & Engedal 2010  (Norway) | Cross-sectional study | 65 Residential Aged Care Facilities | Convenience sample  Survey (response rate not stated)  Descriptive analysis, Logistic regression with multilevel modelling | 623 Residents (Dementia Unit)  Age (Mean)=84 years (sd. 8.6)  Sex=73.5% female  1320 Residents (Regular care)  Age (Mean)=86 years (sd. 8.9)  Sex=68.5% | Dose form modification | 10% of residents were given an inappropriately altered medication, 23% had at least one medication administered in food/beverages. Severe dementia (Adj OR 2.3, *p*=0.003) and low function in ADL (Adj OR 1.2, *p*<0.001) associated with altered medication. |
| Lane et al., 2014  (USA) | Cross-sectional study | 203 Skilled Nursing Facilities | Purposive sample  Secondary dataset (Errors database)  Descriptive analysis, Binomial regression models, Incidence rate ratio estimates, Multivariate logistic regression models | 581 Medication errors from 138 Skilled Nursing Facilities | Medication error | Medication error rate of 3.59 per 100 beds in the first 7 days of admission. Most common type of error was dose omission (36%), or wrong dose (20%). Omissions less likely to cause harm compared to wrong dose (OR = 0.45, 95%CI 0.23-0.89). Larger sites found to have over twice the rate of errors versus sites >100 beds (Model 1, IRR =2.12, Model 2, IRR = 2.11). |
| **Reference** (Country) | **Methodology** | | | ***Participants***  *(N, age, % female)* | **Study focus** | **Key findings** |
|  | Design | Setting | Method |  |  |  |
| Lee et al., 2015  (Hong Kong) | Cross-sectional study | 3 Residential Aged Care Facilities | Convenience sample  Observation  Descriptive analysis, Chi-square test | 304 Medication Administration rounds | Medication administration challenges | Rate of 7.2 interruptions per hour. Average interruption lasted 1-minute, main source was residents (44.0%). Secondary task resulting from interruption usually a direct care task (53%) done immediately (62%). |
| McCloskey et al., 2015 (Canada) | Cross-sectional study (Work flow design) | 7 Residential Aged Care Facilities | Stratified purposive sample  Observation  Descriptive analysis, Chi-square test, Bonferroni correction method (p-values) | 117 Registered Nurses  Age = not stated  Sex = not stated  196 Licenced Practice Nurses  Age = not stated  Sex = not stated  402 Resident Aids  Age = not stated  Sex = not stated  219 Residents  Age (Mean) = 83 years (sd. 12)  Sex = 69% female | Staffing, Time | Medication preparation and administration was a defined care category. Registered nurses spent more time conducing indirect care activities (42.8% in the day and 38.4% in the evening), with the most variation across sites noted around administration of medications and feeding residents. |
| McGillicuddy et al., 2016  (Ireland) | Cross-sectional study | 1 Residential Aged Care Facility | Purposive sample  Retrospective Audit  Descriptive analysis, Chi-square test, Cohen’s Kappa coefficient | 111 Residents  Age (Mean) = 83 years (sd. 7.8)  Sex = not stated | Dose form modification | 77.5% no evidence of swallowing assessment. Medications modified significantly more for continuing care residents (53.7%) vs. respite care (24.3%) (x^2^ yates (1)=8.542, *p*<0.05). Having more medications not significant in relation to dose modification (*p*=0.551). 64.7% of modification done by unlicensed staff, against best practice guidelines. |
| Mercovich, Kyle, Naunton 2014  (Australia) | Cross-sectional study | 2 Residential Aged Care Facilities | Convenience sample  Observation, Survey  Descriptive analysis | 160 Residents  Age = not stated  Sex = not stated | Dose form modification, Education | 18% of residents had at least one medication dose form altered, average of 2.6 medications per resident. More common in Dementia units than high dependency units (21%, 56% versus 7.5%, 6%). 31% of crushed medication were not appropriate (guidelines) |
| Mitty 2009  (USA) | Cross-sectional study | 506 Assisted Living sites | Purposive sample  Survey  Descriptive analysis | *No demographic details given* | Medication error, Staffing | 54% of sites indicated that 80-100% of their residents have their medications administered. 69% of sites used medication aids (staff type). 90% used medication aids to 'assist' rather than administer. Common errors: wrong dose (15%), wrong time (20%). |
| Nicholson & Damons 2022a, 2022b  (South Africa) | Cross-sectional study | 28 Residential Aged Care Facilities | Stratified sampling with randomisation  Surveys (100% response rate; 61% response rate)  Descriptive analysis, Chi-square test, Spearman’s Rho 2-tailed test, Inferential analyses | 123 Nurses  Age (Mean) = 51 years (range of 22-77 years)  Sex = 100% female | Medication administration challenges, Medication error, Staffing | Registered Enrolled nurses primarily gave daytime medication, nursing assistants used overnight. Errors: medication sharing between residents (83% of participants), omission (64.8%), not signing off post-administration (57%), and ‘wrong time’ (50.8%). Key concerns: interruptions (75.6%), stress (42.3%), staffing (39%). ‘Forgetting’ cited as reason for incomplete documentation (81%) |
| **Reference** (Country) | **Methodology** | | | ***Participants***  *(N, age, % female)* | **Study focus** | **Key findings** |
|  | Design | Setting | Method |  |  |  |
| Paradiso et al., 2002  (Australia) | Cross-sectional study | 10 Residential Aged Care Facilities | Stratified sample  Observation  Descriptive analysis, Chi-square tests | 586 Residents  Age = not stated  Sex = not stated  1207 Occasions of medication administration | Dose form modification | 34% of observations involved dose form alteration. 946 oral medications given crushed or opened - 17% were not safe to alter. Sites delivering high level care were more likely to use this practice (x^2^=52.4, df=2, *p*<0.005). 61% of cases involved multiple medications crushed together. 94% of altered doses given in a medication cup mixed with media. |
| Picton et al., 2021  (Australia) | Longitudinal study | 10 Residential Aged Care Facilities | Purposive sample  12-month longitudinal, retrospective audit  Descriptive analysis, Chi-square tests, Poisson model | 392 Residents  Age (Median) = 84 years (range of 76-90)  Sex = 69% female | PRN medications | 93% of residents were administered PRN medication, mean of 5.85 per person-month. Laxatives more commonly prescribed (59%) but opioids most administered (mean 1.54 admins per person-month). Of those who had an opioid or benzodiazepine PRN, 76-77% were administered one of these at least once during the timeframe. |
| Pierson et al., 2007  (USA) | Cross-sectional study | 25 Residential Aged Care Facilities | Convenience sample  Survey, Audit of records  Descriptive analysis | 631 Medication error reports | Medication error | 92% of sites had error reports in the system. Dose omissions most common error type (32%). 67% of serious errors occurred during administration. Licenced practical nurses most implicated in errors (59%). |
| Prasanna et al., 2016  (Sri Lanka) | Cross-sectional study | 9 Residential Aged Care Facilities | Convenience sample  Structured interviews, Records audit, Observation  Descriptive analysis | 100 Residents  Age (Mean) = 70 years (sd. 10.5)  Sex = 72% female | Medication Errors, Self-administration | Mean admin errors per resident was 0.95 (sd. 1.5, median 0.00 (0.00-1.00)), medication omission was the most common error type (50.5%). Of the 45 residents who experienced a medication error, 40% were self-administering. |
| Qian, Yu, & Hailey 2015  (Australia) | Time-motion study | 1 Residential Aged Care Facility | Convenience sample  Observation Time-motion, document review  Workflow mapping, Z- test | 7 Staff  Age=not stated  Sex=86% female | Technology (Electronic medication records) | No significant difference found between paper and electronic records for documentation (*p=0*.011) or administration (*p*=0.607). E-records forced compliance with documentation (time stamped), reduced omissions, and improved recording of medication times. |
| Qian, Yu, & Hailey 2016a  (Australia) | Time-motion study | 1 Residential Aged Care Facility | Convenience sample  Observation, Structured field notes  Descriptive analysis, Mann-Whitney U test, ANOVA, Bonferroni correction. | 7 Staff  Age=not stated  Sex=86% female | Dose form modification, Medication administration challenges, Time | Medication rounds took nurses between 2.5-4.5 hours, an average of 200 seconds per resident. Mixing/crushing medications took significantly longer than not (56 seconds vs 24 seconds). Residents taking tablets themselves shortened time (30 seconds vs 45 seconds with help). |
| Qian, Yu, & Hailey 2016b  (Australia) | Time-motion study | 1 Residential Aged Care Facility | Convenience sample  Observation  Content validity ratio, Z-test | 7 Staff  Age = not stated  Sex = 86% female | Time | Per 8-hour shift, nurses spent between 22.7 and 29.4% of their time on medication administration. Medication administration peaked at 7-9am and 12-1pm (rounds). Staff spent over 25% of their time on medication administration. |
| **Reference** (Country) | **Methodology** | | | ***Participants***  *(N, age, % female)* | **Study focus** | **Key findings** |
|  | Design | Setting | Method |  |  |  |
| Raban et al., 2020  (Australia) | Cross-sectional study | 66 Residential Aged Care Facilities | Convenience sample  Retrospective audit  Descriptive analysis, Generalized estimating equations modelling, Exchangeable correlation matrix | 4787 Residents  Age (Mean) = 85 years (sd. 8)  Sex = 69% female | Technology (Electronic medication records) | Transdermal patches used by 19.6% of residents, with manually entered reminders used for 47.9% of residents (reminders to check the patch). No resident or site characteristics associated with having a reminder to remove patches prior to applying a new patch, but residents of regional sites were more likely to have a check reminder in the electronic record (Adj OR 4.72, CI 1.69-13.20) |
| Roberts et al., 1998  (Australia) | Cross-sectional study | 15 Residential Aged Care Facilities | Random sample  Cross sectional survey  Descriptive analysis, Two-way ANOVA, Multiple linear regression | 1022 Residents  Age (Mean) = 84 years (range of 38 to 105 years)  Sex = 71% female | Medication use, PRN medication | 998 residents had full records available, mean of 6.57 prescribed medications (CI 6.35-6.10; range of 0-22). Mean administered medications, 4.75 (CI 4.56-4.94; range 0-18). 7.8% had no medications administered, 1.6% had none prescribed. PRN medications made up 31% of all prescriptions, 41% of were given at least once during the timeframe. |
| Santos et al., 2016  (UK) | Cross-sectional study | 6 Residential Aged Care Facilities | Convenience sample  Observation  Descriptive analysis, Fisher’s exact test | 166 Residents  Age = not stated  Sex = not stated | Medication error, Medication Omission | 300 administration errors observed, across 100 residents. Wrong administration time was the most common error (126 cases). ‘Wrong time’ error rate per resident 22.06%, omission error rate per resident 13.97%. 58% of omissions were clinical decisions. |
| Scott-Cawiezell et al., 2007  (USA) | Cross-sectional study | 5 Residential Aged Care Facilities | Convenience sample  Observation (Naïve), Document review  Descriptive analysis, Linear modelling, Cochran-Mantel-Haenszel procedure | 39 Staff  Age = not stated  Sex = not stated | Medication error | 97% of 3,194 doses were administered. An average round involved giving 73 medications (range of 8-260), and took on average 113 minutes (25-245 minutes). Registered Nurses administered 15.31% of the doses, medication technicians/aides 61.43% of doses. The error rate for Registered Nurses was 34.6%, technicians/aids 34.2%. Significant relationship between interruptions and errors (*p*=0.0099), |
| Sefidani Forough et al., 2020  (Australia) | Cross-sectional study | 4 Residential Aged Care Facilities | Voluntary sample  Observation  Descriptive analysis, Chi-square test, Students t-test | 12 Staff  Age = not stated  Sex = not stated | Dose form modification | Mean duration of medication prep per resident was 37.9 seconds vs. 11.4 seconds for those that had at least one dose form altered (*p*<0.05, *t*=-7.023). 25.7% of doses were modified, most commonly crushed via a manual crushing device (71.6%). Food was the most common vehicle for doses (66.8%). 12.5% of modifications were inappropriately crushed. |
| Sefidani Forough et al., 2021  (Australia) | Cross-sectional study | Residential Aged Care Facilities (across Australia) | Convenience sample  Survey (sample size calculation of 270 responses, 90% confidence)  Descriptive analysis, Chi-square test, Content analysis (free-text) | 355 Staff  Age = not stated  Sex = not stated | Swallowing difficulty, Dose form modification | 31.3% of respondents were Registered nurses. Overall, 90.9% of respondents had everyday encounters with residents who had swallowing difficulties, most common response method was dose form modification (94.1%). Other reasons included behavioural challenges (63.5%). Barriers: time, workload. Facilitators: support from other staff and professionals, medication reviews. |
| **Reference** (Country) | **Methodology** | | | ***Participants***  *(N, age, % female)* | **Study focus** | **Key findings** |
|  | Design | Setting | Method |  |  |  |
| Seifert & Johnston 205  (USA) | Cross-sectional study | Residential Aged Care Facilities in Texas | Convenience sample  Survey (7.8% response rate)  Descriptive analysis, Mann-Whitney U test, Kruskal-Wallis test, ANOVA, Chi-square test. | 1278 Nurses  Age = not stated  Sex = not stated | Catheters, Dose form modification | Survey focus: Enteral Feeding Catheters (EFCs). Significant differences between rural and urban-sites, urban facilities had more residents that used EFCs (6.6% vs 9.0%, *p*<0.0001), and higher number of oral medications per day (8.3 vs 9.4, *p*=0.0003). |
| Sharma et al., 2021  (Australia) | Cross-sectional study | 8 Residential Aged Care Facilities | Convenience sample of secondary data  Descriptive analysis, Wilcoxon signed rank and McNemar’s tests, Logistic regression, Multivariate logistic regression | 211 Residents  Age (Median) = 87 years (range of 81-92 years)  Sex = 74% female | PRN medications | Paracetamol was the most common PRN medication (54.1% of residents), followed by laxatives (40.9%) and metoclopramide (26.8%). PRN medication accounted for 0.9% of all med admin, and was more likely in regional areas (Adj OR 3.01, 95%CI 1.26-7.19, *p*=0.013). Significant relationship between PRN medication use and Dementia diagnosis (OR 0.56, 95%CI 0.32-0.99, *p=*0.046). |
| Solberg et al., 2021  (Norway) | Cross-sectional study | 8 Residential Aged Care Facility wards | Convenience sample  Observation, Retrospective review of dispensing records  Descriptive analysis, Chi-square test, Regression analysis | 100 Residents  Age (Mean) = 84 years (sd.8.8)  Sex = 53% female | Dose form modification | 20.5% administration episodes involved dose modifications. Of these 80.4% involved crushing. Common reason was swallowing difficulties (53.6%), or lack of resident understanding (19.6%). Significant association between cognitive impairment and dose modification (OR 2.72, *p*=0.003), being female (OR 1.92, *p*=0.034). 10.7% of episodes not in line with guidelines. 69.6% of episodes not noted on medication record. |
| Stasinopoulos et al., 2018  (Australia) | Cross-sectional study | 6 Residential Aged Care Facilities | Convenience sample  Secondary analysis of prior cross-sectional dataset  Descriptive analysis, Univariate logistic regression modelling, Multivariate stepwise logistic regression analysis | 383 Residents  Age (Median) = 88 years (IQR 84-92)  Sex = 78% female | PRN medications | 28% of residents with PRN medication charted had at least one given. The most common medications were analgesics, laxatives. Residents with a larger number of regular medications more likely to be administered PRN medication (Adj OR per additional medication 1.06, 95%CI 1.00-1.13, *p*=.042). Residents with more dependence for activities of daily living were more likely to be administered PRN medications (Adj OR per additional point on the Katz Activities of Daily Life scale: 0.80, 95%CI 072-0.89, *p*<.001). |
| Stokes, Purdie, & Roberts 2004  (Australia) | Cross-sectional study | 13 Residential Aged Care Facilities | Random sample  Medication chart review  Descriptive analysis, Kruskal-Wallis, Mann-Whitney U tests, Spearman’s Rho Correlation. | 801 Residents  Age (Mean) = 84 years (range 80-90 years)  Sex = 73% female | PRN medications | 83.5% of residents had PRN medication available, but only 54.8% of residents received a dose during the timeframe. Analgesics most common (30%), then laxatives (26%) and psycholeptics (17%). Higher use observed in recently hospitalized residents. |
| **Reference** (Country) | **Methodology** | | | ***Participants***  *(N, age, % female)* | **Study focus** | **Key findings** |
|  | Design | Setting | Method |  |  |  |
| Szczepura, Wild, & Nelson 2011  (UK) | Prospective Cohort Study | 13 Residential Aged Care Facilities | Convenience sample  Questionnaire (100% response rate), Secondary data analysis, Observation  Descriptive analysis, Mann-Whitney U test | 345 Residents  Age = not stated  Sex = not stated  45 Staff  Age = not stated  Sex = not stated | Medication error | Average of 206 medication administration episodes per resident per month. 90% were exposed to at least one error, the most common was 'wrong time' (45% of errors); in any one week, 30-39% of residents were at risk of error. Error incidence rate 1.43 (95% CI 1.32-1.56, *p*<0.001). Interruptions noted by 96% of staff surveyed as a key reason for error. |
| Tangiisuran et al., 2018  (Malaysia) | Cross-sectional study | 26 Residential Aged Care Facilities | Convenience sample  Survey (94% response rate)  Descriptive analysis, Chi-square test, independent sample t-test, Fisher exact test. | 155 Staff  Age = 41% between 40-59 years  Sex = 70% female | Education, Staffing | Registered Nurses knowledge scores higher than caregivers (mean score of 12.4 (sd. 1.7) versus 4.5 (sd.3.8), *p*<.001), also for practice (65.2 (Sd. 8.5) versus 40.3 (sd. 10.9), *p*<.001) and attitudes (41.5 (sd. 4.8) vs. 30.8 (sd. 7.3), *p*<0.001). Caregivers had significantly higher stress (*p*=0.002*)*, anxiety (*p*=0.003*)*, and depression (*p*=0.001*)* scores*.* |
| van den Bemt et al., 2009  (Netherlands) | Prospective cohort study | 3 Residential Aged Care Facilities | Convenience sample  Observation  Descriptive analysis, Multivariate analysis | 127 Residents  Age (Median) = 84 years (Range 66-102)  Sex = 72% female | Medication error | 21.2% of administrations were errors. Most common, wrong technique (73%) (crushing medication) followed by wrong time (18% of errors). *Risk factors identified*: being female (OR 1.39, 95%CI 1.05-1.83), antibiotics (OR 11.11; 95%CI 2.66-46.50), crushed medication involved (OR 7.83; 95%CI 5.40-11.36) number of dosages per day per resident (OR 1.03; 95% CI 1.01–1.05), morning doses (OR 2.28; 95% CI 1.50 –3.47), and morning/lunch dose times between 10am and 2pm (OR 1.96; 1.18 –3.27). |
| Vander Stichele et al., 1992  (Belgium) | Cross-sectional study | 20 Residential Aged Care Facilities | Random sample (from a larger convenience sample)  Survey, Interviews, Medication chart review  Descriptive analysis | 198 Residents  Age (Mean)= 83 years (sd. 7)  Sex = 76% female | Resident involvement | 85% of residents had no autonomy over their medication management (16% had it mixed in food). Of those who could consent to an interview (n=128), 81% recalled the dosage of their medications, 71% had knowledge of the indications. Sources of medication information included nursing personnel and family doctors |
| Verrue et al., 2011  (Belgium) | Cross-sectional study | 76 Residential Aged Care Facilities | Representative sample  Surveys, Interviews  Descriptive analysis | 76 Facility Directors  Age = not stated  Sex = not stated  112 Head Nurses  Age = not stated  Sex = not stated | Dose form modification, Medication error | 88.2% of sites had a quality coordinator, 69.7% of sites used a self-reporting error system. 67% noted that medications were administered by care aides. 99.1% reported medications were crushed as needed, justification was that this was due to swallowing issues. 21.4% of nurses referred to guidance before crushing medication. |
| Wright 2002  (UK) | Cross-sectional study | Residential Aged Care Facilities (not specified) | Convenience sample  Survey questionnaire (70.8% response rate)  Descriptive analysis | 540 responses from Nursing staff  Age= not stated  Sex = not stated | Dose form modification | 83.6% of respondents employed in Residential Aged Care Facilities. Most common workarounds for swallowing difficulties: obtaining liquid alternative (87.6%), mix medication with food (56.5% of respondents). 83.8% of respondents had needed to crush tablets/open capsules in the past 12 months |
| **Reference** (Country) | **Methodology** | | | ***Participants***  *(N, age, % female)* | **Study focus** | **Key findings** |
|  | Design | Setting | Method |  |  |  |
| Zimmerman et al., 2011  (USA) | Cross-sectional study | 11 Assisted Living sites | Stratified random sample for sites; Purposive sample for staff  Observation, Document review, Interviews, Survey  Descriptive analysis, Poisson regression | 36 Staff  Age = 50% over the age of 39 years  Sex = 97% female  301 Residents  Age (Mean) = 82 years (sd. 11)  Sex = 69% female | Medication error | 54% of medications were administered in the morning. Overall, 11% of medication 'passes' were interrupted, and 42% of medication passes included an error. 7% of errors were assessed as having moderate-high potential for harm. The odds of an error by staff with less training was double (OR=2.10, 95%CI 1.27-3.49) (non-nurses versus qualified staff). The odds of an error at for-profit sites was three times as high as that in non-profit (OR= 2.89, 95%CI, 1.75-4.00, *p*<0.001). |

**Mixed-Methods Studies (N=10)**

| **Reference** (Country) | **Methodology** | | ***Participants***  *(N, age, % female)* | **Study focus** | **Key findings** |
| --- | --- | --- | --- | --- | --- |
|  | Setting | Method |  |  |  |
| Barber et al., 2009  (UK) | 55 Residential Aged Care Facilities | Random sample  Ethnographic field notes, Observation, Interviews, Audit  Descriptive analysis, Chi-square test, Multi-level modelling | 256 Residents  Age (Mean)= 85 years  Sex = 69% female | Medication error | 22.3% of residents experienced an administration error. Of these, 49% were omissions, and 21.6% were ‘wrong dose’. Patient factors include patients fear and lack of awareness of medicines, medication missing, staff knowledge, and timing around food. Communication was verbal, staffing issues in the AM round, interruptions |
| Breen, Williams, & Wroth., 2023  (Australia) | Residential Aged Care Facilities (number unspecified) | Purposive sample  Retrospective audit of complaints records  Descriptive analysis, Content analysis | 1,134 Complaints related to medication use | Complaints, Medication error | 45% of complaints related to medication administration processes. Categories within this included: 'wrong time', medication management systems not being adequate, resident refusal, and chemical restraint. There was limited opportunity for residents/families to be involved or engaged with around medication management. |
| Dilles et al., 2011  (Belgium) | 20 Residential Aged Care Facilities | Purposive sample of experts; Random sample of sites  Survey (response rate 67% for Registered Nurses, 46% for Nurse Assistants), Expert meeting  Thematic analysis, Descriptive analysis, Chi-square test | *Expert group*  12 Registered Nurses  Age = not stated  Sex = 75% female  *Survey*  246 Registered Nurses  Age (Mean) = 40 years (sd. 9)  Sex = 96.3% female  270 Nurse Assistants  Age (Mean) = 39 years (sd. 11.0)  Sex = 88.6% female | Medication administration challenges, Staffing | 98% of Registered Nurses involved in medication administration, 87% of Nurse Assistants. Barriers to safe medication management included lack of knowledge, time pressure, staff shortages, interruptions, insufficient systems/ guidelines, communication issues, right to refuse, capacity of residents/family to understand medication. |
| Hilleary & Ferrini 2011  (USA) | 1 Residential Aged Care Facility | Unspecified sampling method  Survey, Semi-structured Interviews  Descriptive analysis | 10 Registered Nurses  Age=not stated  Sex=not stated  67 Residents  Age (Mean)=56 years for females, 51 years for males  Sex=42% female | Resident involvement | 92% of residents preferred flavoured water when taking medications and 70% of staff. The product gave the residents an opportunity for decision-making, improved hydration, costed less than juice, had little effect on weight, and was a source of sensory stimulus. |
| Kemp, Luo, & Ball., 2012  (USA) | 45 Assisted Living sites | Random sample  Survey, Semi-structured Interviews  Descriptive analysis, Grounded theory approach/Open coding. | 370 Direct care workers  Age=not stated  Sex=not stated  44 Administrators  Age=not stated  Sex=not stated | Education, Medication administration challenges, Staffing | 75.5% of sites used job shadowing as the only training for medication administration/management. Reluctance to administer medication reported, fear of error. Some staff only allowed to hand package or medications to residents, vs. giving directly by putting medications into residents’ mouths. Emphasis on safety and resident compliance with medication. |
| Lim et al 2016  (UK) | 7 Residential Aged Care Facilities | Purposive sample  Observation, Document review, Interviews  Abstraction hierarchy model, Thematic analysis | 4 Subject matter experts  Age=not stated  Sex=not stated | Medication error | Medication administration identified as one purpose-related function. Requires coordination and input from professionals outside the care home. 9 medication errors identified, stemming from: communication problems, record-keeping issues, and administration issues (wrong medication given, omission). |
| **Reference** (Country) | **Methodology** | | **Participants**  *(N, age, % female)* | **Study focus** | **Key findings** |
|  | Setting | Method |  |  |  |
| Mahmood, Chaudhury, & Gaumont., 2012  (Canada) | 4 Residential Aged Care Facilities | Purposive sample  Observation, Cross-sectional survey (response rate 100%), Focus groups  Descriptive analysis | 54 Nurses  Age=65.3% between 36-55 years  Sex=92.6% Female | Medication error | Interruptions common source of error during medication administration (from staff, residents). Workload, training, and staffing were concerns. Most common error type was omissions (33.3% indicated frequent occurrence). |
| Scott-Cawiezell et al., 2009  (USA) | 5 Residential Aged Care Facilities | Convenience sample  Observation, Secondary data analysis, Document review  Descriptive analysis | 3,700 Residents  Age=not stated  Sex=not stated | Technology (Electronic medication records) | The most common medication error types were late doses, or omissions. Electronic medical records allowed staff to increase the number of medications they gave per hour during a medication round from 40 to an average of 57. Punitive approach to omissions and errors led to poor reporting. |
| Sikma et al., 2014  (USA) | 15 Assisted Living sites | Maximum variation sample  Focussed interviews, Survey (68% response rate, subset of interviewees)  Descriptive analysis, ANOVA, Tukey’s post-hoc test, Constant comparative analysis | 32 Unlicensed Assistive Personnel  Age (Mean)=39 years (sd.12.5)  Sex=97% female  18 Nurses  Age (Mean)=49 years (sd.9.6)  Sex=100% female  8 Pharmacists  Age (Mean)=48 years (sd.11.5)  Sex=36% female  17 Administrators  Age (Mean)=46 years (sd.8.9)  Sex=77% female  9 Primary care physicians  Age (Mean)=50 years (sd.5.3)  Sex=56% female  27 Residents  Age (Mean)=81 years (sd.5.5)  Sex=78% female | Resident involvement, Staffing | Total satisfaction with medication management was statistically significant amongst groups (F(3,77) = 10.21, *p*<0.000), external professionals more negative than all other groups (*p*<0.05). Residents either feared requesting medication assistance (loss of autonomy) or felt unburdened; ability to ask questions/voice concerns about medications was important. Unlicensed assistive personnel described medication administration as demanding, supported required to manage resident refusals and incidents. RN role was to delegate and supervise. |
| Vermeulen et al., 2017  (Netherlands) | 10 Residential Aged Care Facilities | Purposive sample  Semi-structured interviews, Review of medication incidents  Grounded theory approach | 10 Health professionals  Age=not stated  Sex=80% female | Medication error, Safety | Three themes related to increase in self-reported incidents and subsequent contradiction with reduced risks. (1) activities to improve medication safety (improvement initiatives, collaboration); (2) reporting of incidents (culture of reporting change, simplification of processes); (3) impact of supervision. In 2010 risks identified in 45% of items assessed (e.g. medication monitoring, handling of errors), down to 17.4%. |

**Qualitative Studies (N=33)**

| **Reference** (Country) | **Methodology** | | ***Participants***  *(N, age, % female)* | **Study focus** | **Key findings** |
| --- | --- | --- | --- | --- | --- |
|  | Setting | Method |  |  |  |
| Barnes et al., 2006  (Australia) | 10 Residential Aged Care Facilities | Purposive sample  Observation, Semi-structured interviews  Thematic analysis | 11 Registered Nurses  Age=not stated  Sex=not stated | Dose form modification, Medication administration challenges | Overarching concern was balancing staff’s drive to administer all medications versus uncertainty around decision-making. Challenges included resident refusal, education levels of staff, resident’s individual needs, time constraints, and alternative dose forms not being available. |
| Bengtsson et al., 2021  (Sweden) | 7 Residential Aged Care Facilities | Convenience sample  Semi-structured interviews  Deductive content analysis | 21 Staff  Age (Median) = 46 (range 27-64 years)  Sex = 90% female | Medication error | More training around medications and delegation desired by staff. Communication challenges led to carelessness when delegating medication tasks, and underreporting of incidents. |
| Carder 2011  (USA) | 3 Assisted Living sites | Maximum variation sample  Observation, Semi-structured interviews  Grounded theory approach | 16 Medication aids  Age (Mean) = 38 years (sd. 8.3)  Sex = 75% female.  47 residents  Age = not stated  Sex = 77% female | PRN medication | The decision to give PRN medications predominantly stemmed from resident requests, or responses to resident symptoms/behaviour (staff using training and experience). Knowing residents was an important aspect of the decision-making process and influenced PRN medication administration. |
| Carder, Zimmerman, & Schumacher 2009  (USA) | 6 Assisted Living sites | Purposive sample  Ethnographic field notes, Observation, Interviews.  Grounded theory approach | 90 Staff  Age=not stated  Sex=not stated  81 Family members  Age=not stated  Sex=not stated  152 Residents  Age=not stated  Sex=not stated | Resident involvement | Residents ability to have input into their care was constrained by internal/external forces and policy. Self-administration of medication was a source of concern for staff, but some residents appreciated the help with their medication regime. Respect for autonomy and preferences seen to have social and medical consequences (positive and negative). |
| Damiaens et al., 2022  (Belgium) | 4 Residential Aged Care Facilities | Purposive sample  Semi-structured interviews  Inductive thematic analysis | 25 Professionals  Age=76% between the ages of 26-55 years.  Sex=72% female | Person-centred care, Resident involvement | Identified drivers, barriers, features, and perceived consequences of resident/caregiver involvement in medication decision-making. Involvement typically initiated by residents/caregivers, staff reluctant and concerned about risk. |
| Damiaens, Van Hecke, & Foulon 2023  (Belgium) | 4 Residential Aged Care Facilities | Purposive sample  Semi-structured interviews  Inductive thematic analysis | 10 Family members  Age=60% under 65 years  Sex=70% female  17 Residents  Age=71% over 81 years  Sex=82% female | Person-centred care, Resident involvement | Four main themes regarding involvement of residents/caregivers during medication administration. (1) behaviours of involvement, communication; (2) resigned attitudes, limited involvement; (3) organisational and personal factors emphasised safety concerns; (4) participants spoke up and took responsibility if they felt something was wrong. |
| Dawud, Kotecho, & Adamek 2022  (South Africa) | 2 Residential Aged Care Facilities | Purposive sample  In-depth Interviews  Thematic analysis | 14 Formal caregivers  Age (Mean)= 30.2 years  Sex = 57% female  6 Key informants  Age (Mean)= 30 years  Sex = 17% female | Medication administration challenges | Staff at one site assisted with medications, at the other they were the administrators of medication. Challenges included: resident behaviour (refusing to take medication on time, or at all), caregiver burden (too many tasks) |
| **Reference** (Country) | **Methodology** | | ***Participants***  *(N, age, % female)* | **Study focus** | **Key findings** |
|  | Setting | Method |  |  |  |
| Ellis et al., 2012  (Canada) | 2 Residential Aged Care Facilities | Purposive sample  Focus groups  Thematic content analysis | 10 Registered Nurses  Age (Mean)= 42.5 years (sd. 2.5)  Sex = 100% female  12 Registered Practical Nurses  Age (Mean)= 39.0 years (sd. 2.3)  Sex = 100% female | Medication administration challenges, Time | Medication management described as a complex race against time. Three themes: preparing to race (knowing the resident and medication, collaborating with care team); running the race (multitasking and strategizing, methods to speed up admin); finishing the race (evaluating residents, documentation). Barriers: time pressure, interruptions, over-documentation, communication issues, knowledge issues, resident refusal of medication). Facilitators: communication, collaboration, staffing, knowledge about resident/medication. |
| Garratt et al., 2021  (New Zealand) | 9 Residential Aged Care Facilities | Purposive sample, Maximum variation sample  Focus groups, Semi-structured Interviews  Thematic analysis, Thematic mapping | 32 Staff  Age=not stated  Sex=not stated  12 Residents  Age=not stated  Sex=not stated | Dose form modification, Medication administration challenges | Four themes about crushing medication: (1) a workaround to ensure compliance; (2) ethically conflicting; (3) had a relational impact (trust and communication concerns); (4) staff lacked knowledge about medications – more pharmacy input was needed. |
| Gilbert & Kim 2018  (Australia) | 1 Residential Aged Care Facility | Sampling method unspecified  Case study  Root cause analysis | 1 Resident  Age=86 years  Sex=0% female | Medication error | Communication about medication changes post-hospital discharge is important, new staff in Residential Aged Care Facilities must be supported to call on more experience staff to verify use/dosage of medications if unsure or concerned. |
| Gilmartin, Marriott, & Hussainy 2014  (Australia) | 49 Residential Aged Care Facilities | Purposive sample of professionals across sites  Focus groups  Thematic analysis, Framework approach | 13 Health professionals  Age=not stated  Sex=not stated | Dose administration aids | Contributing factors to dose administration aid (sachets or blister packs) incidents. (1) unreliable communication between professional groups; (2) lack of knowledge/ education around dose administration aid preparation; (3) medication handling issues; (4) poor attitude towards incidents. Solutions: more open collaboration, better preparation, knowledge sharing,multi-disciplinary meetings. |
| Gilmartin, Jani, & Smith 2015  (UK) | Residential Aged Care Facilities (number unspecified) | Purposive sample  Semi-structured interviews  Thematic analysis | 8 Expert pharmacists  Age=not stated  Sex=75% female | Dose administration aids | Dose administration aids/compliance aids seen as a safer method for medication administration (pre-packed for each resident, reduces time, simplifies process), and good for those who self-administer. Noted that these aids could lead to a decline in staff alertness, detachment from clinical monitoring. |
| Gransjon Craftman et al., 2016  (Sweden) | Residential Aged Care Facilities (number unspecified) | Purposive sample  Semi-structured interviews  Manifest content analysis | 18 Registered Nurses  Age (Range)=38-66 years  Sex=94% female | Staffing | Delegation to unlicensed staff was challenging, unlicensed staff required more training. Common deviations noted as missed doses, not signing off post administration, wrong doses given, incorrect administration. Trust, communication were lacking, created uncertainty in delegation situations. |
| Hughes et al., 2009  (Northern Ireland) | Residential Aged Care Facilities (number unspecified) | Purposive sample  Semi-structured Interviews, Focus groups  Constant comparison, Grounded theory | 8 General Practitioners  Age =not stated  Sex =36% female  9 Registered Nurses  Age =not stated  Sex =100% female  17 Residents  Age (Mean) =81 years (sd. 11)  Sex =53% female | Resident involvement | Tension between resident autonomy/staff control. Poor documentation, polypharmacy, adverse effects of medication were issues. Residents refused medication for fear of adverse effects. Staff recognized residents right to have input into care, tempered by perceptions of residents' ability to have input, logistical factors. |
| **Reference** (Country) | **Methodology** | | ***Participants***  *(N, age, % female)* | **Study focus** | **Key findings** |
|  | Setting | Method |  |  |  |
| Karsan et al., 2021  (UK) | 3 Residential Aged Care Facilities | Purposive sample  Semi-structured interviews  Thematic analysis, Thematic mapping | 15 Staff  Age=not stated  Sex=not stated | Medication administration challenges, Technology | Four groups of barriers: infrastructural, operational (safety and processes), implementation team, and system user barriers). Staff attitudes/ engagement was a concern across barrier types. Electronic chart sustainability required co-development, on-going training, to align with current legislation. |
| Kuppadakkath, Olasoji, & Garvey 2022  (Australia) | Residential Aged Care Facilities | Convenience non-probability, snowball sampling  Semi-structured interviews  Thematic analysis | 12 Registered Nurses  Age= 92% between 21-40 years  Sex=not stated | Medication error | Four overarching themes. (1) Environment concerns; (2) External constraints e.g. delays, interruptions; (3) psychological impact of errors on staff, residents, and families; (4) strategies to prevent medication error ('do not disturb' signs identified as a strategy, electronic reminders, more regular staff vs agency staff per shift). |
| Lei et al., 2023  (Macau) | 9 Residential Aged Care Facilities | Purposive sample  Semi-structured interviews  Thematic analysis | 57 Staff  Age=not stated  Sex=75% female | Technology (Electronic medication records) | Six themes: (1) perceived expectations: reduce errors/improve safety;(2) input: funding and investment needed; (3) medication activities/ recording change to real-time electronic;(4-5) benefits: error reduction, better communication; (6) adoption factors: pharmacy input, internet coverage, tech support, systems disconnect. |
| Motta et al., 2018  (Brazil) | 1 Residential Aged Care Facility | Purposive sample  Interviews, Photo elicitation, Observation  Inductive content analysis | 6 Staff  Age=Range of 36-50 years  Sex=not stated | Medication error | Four themes: (1) work design/workplace reduces error risk and improves safety; (2) training is essential for safe medication administration; (3) good communication is essential; (4) without a patient safety culture it is difficult for staff to grow and learn (e.g. fear of reprisal if errors reported) |
| Oates et al., 2019  (UK) | 10 Residential Aged Care Facilities | Purposive sample  Semi-structured Interviews  Thematic analysis | 10 Residents  Age (Mean)=83 years (sd.7.1)  Sex=60% female | Medication administration challenges, Resident involvement | Some residents preferred control of their medication, others felt relieved of a burden if staff were responsible. Dose times important for quality of life, functional ability. Residents wanted to be believed when they noted concerns, not dismissed. |
| Odberg et al., 2018  (Norway) | 2 Residential Aged Care Facilities | Purposive sample  Observations  Inductive content analysis | 16 Residents  Age=not stated  Sex=not stated  54 staff  Age=not stated  Sex=not stated | Medication administration challenges | Medication administration was a complex series of processes, with high levels of interprofessional collaboration. Complexity and interruptions considered normal. Interruption types: active (break from task to complete another); Passive (stimuli based; Tech (e.g. alarms, login process, internet). |
| Odberg, Hansen, & Wangensteen 2019  (Norway) | 2 Residential Aged Care Facilities | Purposive sample  Observations, Semi-structured interviews  Inductive content analysis | 13 Nursing staff  Age=not stated  Sex=80% female | Medication administration challenges | Three categories for nurses role: (1) Compensating (Individual level): impacted by the competence of other staff, documentation prioritized; (2) Flexible (team level): distributed leadership, delegation limited by competence;(3) Adaptable (organisational level): shifts planned to balance competencies and continuity of care. |
| Odberg et al., 2020  (Norway) | 1 Residential Aged Care Facility | Convenience sample  Observations, interviews  Deductive content analysis, Work system analysis | 9 Nursing staff  Age=not stated  Sex=80% female | Safety, Technology (Electronic medication records) | Facilitators for safe administration included electronic medication records, consistent staff, communication, staff competence, resident involvement. Barriers included physical environment, interruptions, technology 'overload' and delays (e.g. login time), noise. |
| **Reference** (Country) | **Methodology** | | ***Participants***  *(N, age, % female)* | **Study focus** | **Key findings** |
|  | Setting | Method |  |  |  |
| Qian et al., 2018  (Australia) | 1 Residential Aged Care Facility | Convenience sample  Observation  Content analysis, Coding framework | 7 Staff  Age=not stated  Sex=86% female | Medication administration challenges | Medication administration is both medication and resident-centric, complex, prone to interruptions. Rapport was important with residents, even under time pressure. Deviations from process included: not using resident’s photo, not signing record post-administration. |
| Reinhard et al., 2006  (USA) | Assisted Living sites (Number unspecified) | Convenience sample  Semi-structured interviews  Descriptive analysis, Content analysis | 42 Board of Nursing Executives  Age=not stated  Sex= not stated | Staffing | 22 states allowed for nurses to delegate medication administration (oral medications primarily). In 18 states unlicensed assistive personnel could only help with self-administration (remind residents). |
| Sawan, Kouladjian O'Donnell, & Hilmer 2020  (Australia) | 4 Residential Aged Care Facilities | Purposive sample  Semi-structured interviews, focus groups  Thematic analysis | 19 Staff  Age (Median)=41 years (range of 25-57)  Sex= 95% female | Resident involvement | Five themes: (1) Staff would devolve responsibility about goals/preferences for medications to the prescriber; (2) Inconsistent documentation; (3) person-centred assessments were lacking; (4) communication gaps between staff and GPs; (5) Staff lacked knowledge about pharmaceutical care goals and resident-orientated outcomes. |
| Sefidani Forough et al., 2020  (Australia) | 3 Residential Aged Care Facilities | Purposive sample  Semi-structured interviews  Quantitative content analysis (Leximancer software) | 17 Staff  Age = not stated  Sex = 100% female | Dose form modification, Swallowing difficulty | Three clusters: (1) work process factors (time pressures, workload, education, staffing stress); (2) medication related factors (how to facilitate administration, dose form modification uncertainty, alternative forms not available, multidisciplinary communication challenges); (3) resident related factors (preferences, needs, dementia). |
| Sharpp, Kayser-Jones, & Young 2012  (USA) | 1 Assisted Living site | Convenience sample  Ethnographic observation, Semi-structured Interviews  In-vivo coding | 20 Staff  Age (Mean) = 43 years (sd. 10)  Sex = 80% female  35 Residents  Age (Mean) = 79 years (sd. 10)  Sex = 83% female | Dementia, Education | Four themes related to care provision: (1) Lack of knowledge about Dementia; (2) Lack of knowledge about acute illnesses (preventing/assessing, infection control); (3) limits in monitoring/reporting resident changes (slow); (4) inappropriate medication administration (omissions, delays). |
| Solberg et al., 2022  (Norway) | 3 Residential Aged Care Facilities | Purposive sample  Focus groups  Qualitative content analysis | 11 Nurses  Age (Mean) = 47 years (sd. 10.5)  Sex = 91% female | Communication, Medication administration challenges, | Overarching theme of “the art of making the right exception to the rule”. (1) Coping with obstacles and opportunities (information flow, professional resources, complex medication regimes); (2) Adapting to patients and contexts (individual needs, keeping pace); (3) Competence, safety (clinical judgement, medication knowledge). |
| Tariq, Georgiou, & Westbrook 2013a  (Australia) | 3 Residential Aged Care Facilities | Purposive sample  Non-participant observation, Semi-structured interviews  Open coding, Triangulation | 3 Quality Management Team Members  Age = not stated  Sex = not stated  130 Residents  Age = not stated  Sex = not stated | Dose administration aids, Technology | *Pre-administration:* prepping medications and administration sheets, checking packs, checking non-packed items. Key hazards included missing short course medication, expiry dates, inaccurate maintenance of records.  *During administration:* checking medications and residents, identifying packing errors. Concerns included short staffing interruptions, timing of medications.  *Post-administration:* verbal handover, progress notes, following up errors, reporting medication refusals and low stock. For refusals, even if only part of the dose, recorded as whole dosage as staff were not qualified to identify meds within the pack. |
| **Reference** (Country) | **Methodology** | | ***Participants***  *(N, age, % female)* | **Study focus** | **Key findings** |
|  | Setting | Method |  |  |  |
| Tariq, Georgiou, & Westbrook 2013b  (Australia) | 3 Residential Aged Care Facilities | Convenience sample  Ethnographic observation, Interviews and informal conversations, Field notes, Photographs  Thematic content analysis | 18 Staff  Age=not stated  Sex=not stated  130 Residents  Age=not stated  Sex=not stated | Medication error | Three information exchange dimensions contribute to medication error occurrence: (1) complicated record keeping and order processing (chart design, unclear information, reviews); (2) lack of coordination mechanisms between parties (pharmacy, prescriber, aged care staff), impacted communication and collaboration; (3) reliance on telephone/fax, complicates information processing requirements. |
| Tariq et al., 2014  (Australia) | 1 Residential Aged Care Facility | Purposive sample  Observation, Semi-structured interviews  Grounded theory approach | 4 Staff  Age=not stated  Sex=not stated  78 Residents  Age=not stated  Sex=not stated | Technology (Electronic medication records) | Site had been using the electronic system for nearly 24 months. Manual aspects still used as part of management process – the electronic system implemented was not end-to-end, prescribing and pharmacy communication still using paper charts. Five challenges identified: (1) limited interactivity within the system design; (2) system not flexible enough; (3) information layout and semantics of system problematic; (4) no decision-making support for staff; (5) system maintenance issues. |
| Vogelsmeier, Scott-Cawiezell, & Zellmer 2007  (USA) | 5 Residential Aged Care Facilities | Purposive sample  Key informant interviews, Focus groups  Thematic analysis | 76 Staff  Age=not stated  Sex=not stated | Medication administration challenges, Safety | Common themes: (1) communication issues between staff; (2) competing demands on staffs' time - which had implications for timeliness; (3) challenges of paper charts which had implications for accuracy e.g. 'stop dates', 'do not crush'. |
| Young et al., 2013  (USA) | 12 Assisted Living sites | Purposive sample  Observation, Semi-structured interviews  Constant comparative analysis | 44 Medication Aides  Age (Mean)=37 years (sd.12.2)  Sex= 96% female  18 Registered Nurses  Age (Mean)=49 years (sd.9.6)  Sex= 100% female  17 Administrators  Age (Mean)=46 years (sd.8.9)  Sex= 77% female  9 Primary Care Providers  Age (Mean)=50 years (sd.5.3)  Sex= 56% female  8 Pharmacists  Age (Mean)=48 years (sd.11.0)  Sex= 38% female | Medication administration challenges, Safety, Staffing | Six key strategies identified to promote medication safety in assisted living. (1) Clear RN oversight; (2) Supported orientation and training of medication aides; (3) Facility policies that respond to local conditions and promote safety; (4) Recognition of timing of administration (e.g. morning medication pass being the most complex, requiring more staff); (5) Identification of high-risk situations (medication or resident-centred risks); (6) Implementing system and individual level communication approaches (reducing interruptions/unwanted communications and increasing cross-shift communication) |
